# An Indexing Theory for Working Memory Based on Fast Hebbian Plasticity

**DOI:** 10.1523/ENEURO.0374-19.2020

**Published:** 2020-03-19

**Authors:** Florian Fiebig, Pawel Herman, Anders Lansner

**Affiliations:** 1 Lansner Laboratory, Department of Computational Science and Technology, Royal Institute of Technology, 10044 Stockholm, Sweden; 2 Department of Mathematics, Stockholm University, 10691 Stockholm, Sweden

**Keywords:** computational model, long-term memory, short-term memory, spiking neural network, synaptic plasticity, working memory

## Abstract

Working memory (WM) is a key component of human memory and cognition. Computational models have been used to study the underlying neural mechanisms, but neglected the important role of short-term memory (STM) and long-term memory (LTM) interactions for WM. Here, we investigate these using a novel multiarea spiking neural network model of prefrontal cortex (PFC) and two parietotemporal cortical areas based on macaque data. We propose a WM indexing theory that explains how PFC could associate, maintain, and update multimodal LTM representations. Our simulations demonstrate how simultaneous, brief multimodal memory cues could build a temporary joint memory representation as an “index” in PFC by means of fast Hebbian synaptic plasticity. This index can then reactivate spontaneously and thereby also the associated LTM representations. Cueing one LTM item rapidly pattern completes the associated uncued item via PFC. The PFC–STM network updates flexibly as new stimuli arrive, thereby gradually overwriting older representations.

## Significance Statement

Most, if not all, computational working memory (WM) models have focused on short-term memory (STM) aspects. However, from the cognitive perspective the interaction of STM with long-term memory (LTM) bears particular relevance since the WM-activated LTM representations are considered central to flexible cognition. Here we present a large-scale biologically detailed spiking neural network model accounting for three connected cortical areas to study dynamic STM–LTM interactions that reflect the underlying theoretical concept of memory indexing, adapted to support distributed cortical WM. Our cortex model is constrained by relevant experimental data about cortical neurons, synapses, modularity, and connectivity. It demonstrates encoding, maintenance, and flexible updating of multiple items in WM as no single model has done before. It thereby bridges microscopic synaptic effects with macroscopic memory dynamics, and reproduces several key neural phenomena reported in WM experiments.

## Introduction

By working memory (WM) we typically understand a flexible but volatile kind of memory capable of holding a small number of items over short time spans, allowing us to act beyond the immediate here and now. WM is thus a key component in cognition and is often affected early on in neurologic and psychiatric conditions (e.g., Alzheimer’s disease and schizophrenia; Slifstein et al., 2015). Although prefrontal cortex (PFC) has consistently been implicated as a key neural substrate for WM in humans and nonhuman primates (Fuster, 2009; D’Esposito and Postle, 2015), there is accumulated evidence for the involvement of other cortical regions, particularly parietotemporal networks associated with long-term memory (LTM) correlates. Consequently, there is growing understanding that WM function emerges from the interactions between dynamically coupled short-term memory (STM) and LTM systems (Eriksson et al., 2015; Sreenivasan and D’Esposito, 2019), which enable activation or the “bringing online” of a small set of task-relevant LTM representations (Eriksson et al., 2015). This prominent effect is envisaged to underlie complex cognitive phenomena, which are reported in experiments on humans as well as animals. Nevertheless, since there is limited availability of multiarea mesoscopic recordings of neural activity during WM, the neural mechanisms involved remain elusive. Furthermore, computational models of WM have so far focused solely on its short-term memory aspects, explained either by means of persistent activity (Funahashi et al., 1989; Goldman-Rakic, 1995; Camperi and Wang, 1998; Compte et al., 2000) or, more recently, fast synaptic plasticity (Mongillo et al., 2008; Lundqvist et al., 2011; Fiebig and Lansner, 2017), and there are no detailed hypotheses about neural underpinnings of the operational STM–LTM interplay in the service of WM.

To address this gap and draw attention to the wider cognitive perspective of WM accounting for more than STM correlates in PFC, we present a large-scale multiarea spiking neural network model of WM and focus on investigating the neural mechanisms behind the fundamental STM–LTM interactions critical to WM function. Our model comprises a subsampled PFC network model of STM that is reciprocally connected with two LTM component networks representing different sensory modalities (e.g., visual and auditory) in parietotemporal cortical areas. This new model exploits the architecture of a recent PFC-dependent STM model of human word-list learning (Fiebig and Lansner, 2017), shown to reproduce a range of patterns of mesoscopic neural activity observed in WM experiments. It uses the same fast Hebbian plasticity as a key neural mechanism, intrinsically within PFC but also in PFC backprojections that target parietotemporal LTM stores. The core idea of our theory rests on the concept of cell assemblies formed in the PFC, as STM correlates, by means of fast Hebbian plasticity that serve as “indices” linking LTM representations. The associative plasticity in this functional context has to be induced and expressed on a timescale of a few hundred milliseconds. Recent experiments have demonstrated the existence of fast forms of Hebbian synaptic plasticity (e.g., short-term potentiation or labile LTP; Erickson et al., 2010; Park et al., 2014; Pradier et al., 2018), which lends credibility to this type of WM mechanism.

The proposed concept of distributed WM resting on the dynamical STM–LTM interactions, mediated by fast synaptic plasticity, draws inspiration from the hippocampal memory indexing theory (Teyler and DiScenna, 1986), originally proposed to account for the role of hippocampus in storing episodic memories (Teyler and Rudy, 2007). Binding and indexing of neural representations have been a common recurring theme in memory research, in particular in relation to the role of hippocampus and surrounding structures (Squire, 1992; O’Reilly and Frank, 2006; Teyler and Rudy, 2007). We therefore adapt this theoretical notion and formulate a cortical indexing theory of WM, thereby reflecting a more general computational principle of indexing that supports multiarea memory phenomena. Our main novel contribution here is to show that a neurobiologically constrained large-scale spiking neural network model of interacting cortical areas via biologically realistic sparse connectivity can function as a robust and flexible multi-item and cross-modal WM. This includes its important role of bringing relevant LTM representations temporarily online by means of “indexing,” and thus to computationally validate the proposed concept of distributed WM. In addition, the model replicates many experimentally observed effects in terms of oscillations, coherence, and latency within and between cortical regions, and offers new macroscopic predictions about large-scale internetwork dynamics as a neural correlate of WM operations. Interestingly, it can also explain the so far poorly understood cognitive phenomenon of variable binding or object–name association, which is one key ingredient in human reasoning and planning (Cer and O’Reily, 2012; Pinkas et al., 2013; van der Velde and de Kamps, 2015).

## Materials and Methods

### Neuron model

We use an integrate-and-fire point neuron model with spike–frequency adaptation (Brette and Gerstner, 2005), which was modified by Tully et al. (2014) for compatibility with a custom-made Bayesian Confidence Propagation Neural Network (BCPNN) synapse model in NEST (see Simulation environment) through the addition of the intrinsic excitability current $I_{\beta_{j}}$. The model was simplified by excluding the subthreshold adaptation dynamics. Membrane potential (*V_m_*) and adaptation current are described by the following equations:

(1) $$-C_{m}\frac{dv_{m}}{dt}=-g_{L}\left(V_{m}-E_{L}\right)+g_{L}\Delta_{T}e^{\frac{v_{m}-v_{t}}{\Delta_{T}}}-I_{w}(t)-I_{tot}(t)+I_{\beta_{j}}+I_{ext}$$

(2) $$\frac{dI_{w}(t)}{dt}=\frac{-l_{w}(t)}{\tau_{I_{w}}}+b\delta \left(t-t_{sp}\right).$$

The membrane voltage changes through incoming currents over the membrane capacitance (*C_m_*). A leak reversal potential (*E_L_*) drives a leak current through the conductance (*g_L_*), and an upstroke slope factor (Δ*_T_*) determines the sharpness of the spike threshold (*V_t_*). Spikes are followed by a reset of membrane potential to *V_r_*. Each spike increments the adaptation current by *b*, which decays with time constant $\tau_{I_{w}}$. Simulated basket cells feature neither the intrinsic excitability current $I_{\beta_{j}}$ nor this spike-triggered adaptation.

In addition to external input *I*_ext_ (see Stimulation protocol), neurons receive a number of different synaptic currents from their presynaptic neurons in the network (AMPA, NMDA, and GABA), which are summed at the membrane accordingly:

(3) $$I_{tot_{j}}(t)=\sum_{syn}\sum_{i}g_{ij}^{syn}(t)\left(V_{m_{j}}-E_{ij}^{sym}\right)=I_{j}^{AMPA}(t)+I_{j}^{NMDA}(t)+I_{j}^{GABA}(t).$$

### Synapse model

Excitatory AMPA and NMDA synapses have a reversal potential *E^AMPA^* = *E^NMDA^*, while inhibitory synapses drive the membrane potential toward *E*^GABA^. Every presynaptic input spike (at $t_{sp}^{i}$ with transmission delay *t_ij_*) evokes a transient synaptic current through a change in synaptic conductance that follows an exponential decay with time constants *τ*^syn^ depending on the synapse type (*τ*^AMPA^ ≪ *τ*^NMBA^), as follows:

(4) $$g_{ij}^{syn}(t)=x_{ij}^{dep}(t)w_{ij}^{syn}e^{-\frac{t-t^{\iota }-t_{ij}}{\tau^{syn}}}H\left(t-t_{sp}^{i}-t_{ij}\right).$$

*H*(·) is the Heaviside step function. $w_{ij}^{syn}$ is the peak amplitude of the conductance transient, learned by the spike-based BCPNN learning rule (next section). Plastic synapses are also subject to synaptic depression (vesicle depletion) according to the Tsodyks–Markram formalism (Tsodyks and Markram, 1997), modeling the transmission-dependent depletion of available synaptic resources $x_{ij}^{dep}$ by a utilization factor *U*, and a depression/reuptake time constant *τ*_rec_, as follows:

(5) $$\frac{dx_{ij}^{dep}}{dt}=\frac{1-x_{ij}^{dep}}{\tau_{rec}}-Ux_{ij}^{dep}\sum_{sp}\delta \left(t-t_{sp}^{i}-t_{ij}\right).$$

### Spike-based BCPNN learning rule

Plastic AMPA and NMDA synapses are modeled to mimic NMDA-dependent Hebbian short-term potentiation (Erickson et al., 2010) with a spike-based version of the BCPNN learning rule (Wahlgren and Lansner, 2001; Tully et al., 2014). For a full derivation from Bayes rule, deeper biological motivation, and proof of concept, see Tully et al. (2014) and an earlier STM model implementation by Fiebig and Lansner (2017).

Briefly, the BCPNN learning rule makes use of biophysically plausible local traces to estimate normalized presynaptic and postsynaptic firing rates, as well as coactivation, which can be combined to implement Bayesian inference because connection strengths and neural unit activations have a statistical interpretation (Sandberg et al., 2002; Fiebig and Lansner, 2014; Tully et al., 2014). Crucial parameters include the synaptic activation trace *Z*, which is computed from spike trains via presynaptic and postsynaptic time constants $\tau_{z_{i}}^{syn},\tau_{z_{j}}^{syn}$, which are the same here but differ between AMPA and NMDA synapses, as follows:

(6) $$\tau_{z_{i}}^{AMPA}=\tau_{z_{j}}^{AMPA}=5ms,\;\tau_{z_{i}}^{NMDA}=\tau_{z_{j}}^{NMDA}=100ms.$$

The larger NMDA time constant reflects the slower closing dynamics of NMDA receptor-gated channels. All excitatory connections are drawn as AMPA and NMDA pairs, such that they feature both components. Further filtering of the Z traces leads to rapidly expressing memory traces (referred to as P-traces) that estimate activation and coactivation as follows:

(7) $$\begin{array}{lll} \tau_{p}\frac{dP_{i}}{dt}=\kappa \left(Z_{i}-P_{i}\right), & \tau_{p}\frac{dP_{j}}{dt}=\kappa \left(Z_{j}-P_{j}\right), & \tau_{p}\frac{dP_{ij}}{dt}=\kappa \left(z_{i}z_{j}-P_{ij}\right). \end{array}$$

These traces constitute memory itself and decay in a palimpsest fashion. Short-term potentiation decay is known to take place on timescales that are highly variable and activity dependent (Volianskis et al., 2015; see Discussion, The case for Hebbian plasticity).

We make use of the learning rule parameter *κ* (Eq. 7), which may reflect the action of endogenous neuromodulators [e.g., dopamine (DA) acting on D_1_ receptors (D1Rs)] that signal relevance and thus modulate learning efficacy). It can be dynamically modulated to switch off learning to fixate the network or temporarily increase plasticity (*κ*_encoding_, *κ*_normal_; Table 1). In particular, we trigger a transient increase of plasticity concurrent with external stimulation.

**Table 1:** Neurons, synapses, and plasticity

Adaptation current	b	86 pA	Depression time constant	*τ*_rec_	500 ms	BCPNN AMPA gain	$w_{gain}^{AMPA}$	3.93 nS
Adaptation time constant	$\tau_{I_{w}}$	500 ms	AMPA synaptic time constant	*τ*^AMPA^	5 ms	BCPNN NMDA gain	$w_{gain}^{NMDA}$	0.21 nS
Membrane capacity	*C_m_*	280 pF	NMDA synaptic time constant	*τ*^NMDA^	100 ms	BCPNN bias current gain	β_gain_	90 *pA*
Leak reversal potential	*E_L_*	−70 mV	GABA synaptic time constant	*τ*^GABA^	5 ms	BCPNN lowest rate	*f*_min_	0.2 Hz
Leak conductance	*g_L_*	14 pS	AMPA reversal potential	*E*^AMPA^	0 mV	BCPNN highest rate	*f*_max_	20 Hz
Upstroke slope factor	Δ*_T_*	3 mV	NMDA reversal potential	*E*^NMDA^	0 mV	BCPNN lowest probability	ε	0.01
Spike threshold	*V_t_*	−55 mV	GABA reversal potential	*E*^GABA^	−75 mV	BCPNN Spike event duration	Δ*t*	1 ms
Spike reset potential	*V_r_*	−80 mV	Dopaminergic modulation	*κ*_encoding_	6.0	P-trace time constant	*τ_p_*	5 s
Utilization factor	*U*	0.33	Regular plasticity	*κ*_normal_	1.0			

Tully et al. (2014) showed that Bayesian inference can be recast and implemented in a network using the spike-based BCPNN learning rule. Prior activation levels are realized as an intrinsic excitability of each postsynaptic neuron, which is derived from the postsynaptic firing rate estimate *p_j_* and implemented in the NEST neural simulator (Gewaltig and Diesmann, 2007) as an individual neural current ${\text{I}}_{\beta_{\text{j}}}$ with scaling constant *β*_gain_:

(8) $${\text{I}}_{\beta_{\text{j}}}=\beta_{\text{gain}}\log\left({\text{P}}_{\text{j}}\right).$$

${\text{I}}_{\beta_{\text{j}}}$ is thus an activity-dependent intrinsic membrane current to the neurons, similar to the A-type potassium channel (Hoffman et al., 1997) or TRP channel (Petersson et al., 2011). Synaptic weights are modeled as peak amplitudes of the conductance transient (Eq. 4) and determined from the logarithmic BCPNN weight, as derived from the P-traces with a synaptic scaling constant $w_{gain}^{syn}$, as follows:

(9) $$w_{ij}^{syn}=w_{gain}^{syn}\log\frac{p_{ij}}{p_{i}p_{j}}.$$

**Figure 1.**
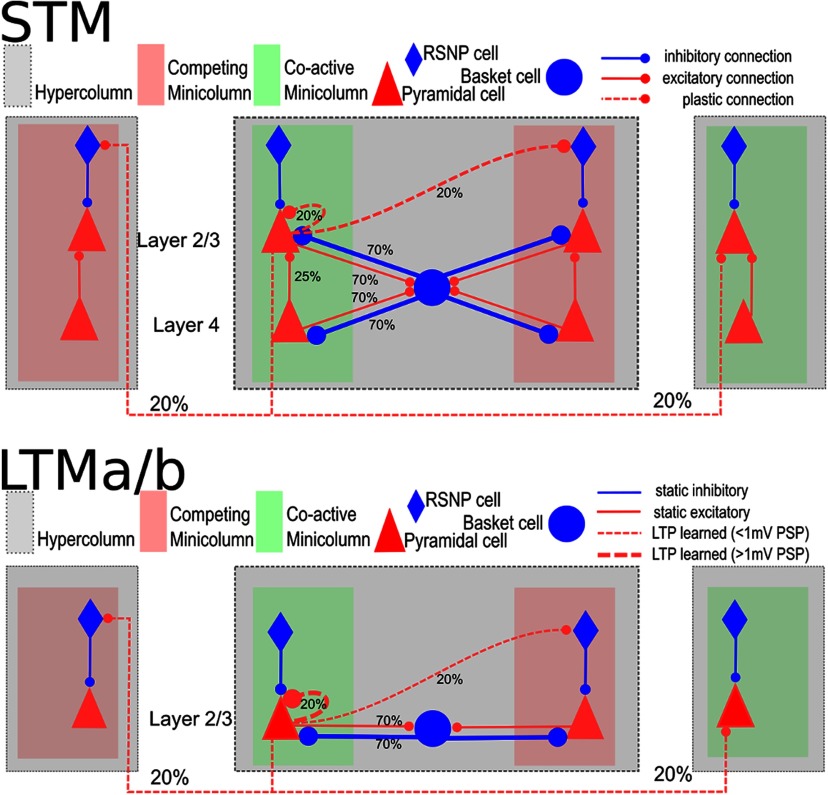
Local columnar connectivity within STM and LTM. Connection probabilities are given by the percentages; further details are in Tables 1, 2, and 3. The strength of plastic connections develops according to the synaptic learning rule described in the spike-based BCPNN learning rule. Initial weights are low and distributed by a noise-based initialization procedure (see Stimulation protocol). However, dashed connections are not plastic in LTM (besides the synaptic depression of Eq. 5), but already encode memory patterns previously learned through an LTP protocol, and loaded before the simulation using receptor-specific weights found in Table 2.

In this model, AMPA and NMDA synapses make use of $w_{gain}^{{}^{AMPA}}$ and $w_{gain}^{NMDA}$, respectively. The logarithm in Equations 8 and 9 is motivated by the Bayesian underpinnings of the learning rule and means that synaptic weights $w_{ij}^{syn}$ multiplex both the learning of excitatory and disynaptic inhibitory interaction. The positive weight component is here interpreted as the conductance of a monosynaptic excitatory pyramidal to pyramidal synapse [Fig. 1, plastic connection to the coactivated minicolumn (MC)], while the negative component (Fig. 1, plastic connection to the competing MC) is interpreted as disynaptic via a dendritic targeting and vertically projecting inhibitory interneuron like a double bouquet and/or bipolar cell (Tucker and Katz, 2003; Kapfer et al., 2007; Ren et al., 2007; Silberberg and Markram, 2007). Accordingly, BCPNN connections with a negative weight use a GABAergic reversal potential instead, as in previously published models of this kind (Tully et al., 2014, 2016; Fiebig and Lansner, 2017). Model networks with negative synaptic weights have been shown to be functionally equivalent to those with both excitatory and inhibitory neurons with only positive weights (Parisien et al., 2008). In the context of this particular model microcircuit and learning rule, this was explicitly and conclusively demonstrated by the addition of double bouquet cells (Chrysanthidis et al., 2019).

**Table 2:** Network size, conduction delay, stimulation, and LTM preload BCPNN weights

STM patch size	17 × 17 mm		Initialization input rate layer 2/3	$r_{bg-low}^{L23}$	550 Hz
Simulated HCs	$n_{HC}^{STM}$	25	Background activity rate layer 2/3	$r_{bg}^{L23}$	625 Hz
Simulated MC per HC	$n_{MC}^{STM}$	12	Background activity rate layer 4	$r_{bg}^{24}$	300 Hz
LTM patch size	25 × 25 mm		High Background activity rate layer 2/3 (e.g., STM maintenance)	$r_{bg-high}^{L23}$	950 Hz
Simulated HCs	$n_{HC}^{LTM}$	16			
Simulated MC per HC	$n_{MC}^{LTM}$	9	Background conductance	*g_bg_*	±1.5 nS
Axonal conduction speed	*V*	2$\frac{m}{s}$			
Minimal conduction delay	$t_{min}^{syn}$	1.5 ms	Cue stimulus duration	*t_cue_*	50 ms
STM–LTM distance	*d_STM-LTM_*	40 mm	Stimulation rate	*r_cue_*	650 Hz
Hypercolumn diameter	*d_HC_*	0.64 mm	Cue stimulus conductance	*g_cue_*	+1.5 nS
Layer 2 pyramidal per MC	$n_{MC}^{PYR-L2}$	20	LTM intra-HC–intra-MC weight	$w_{IntraMC}^{IntraHC}$	3.36 $w_{gain}^{syn}$
Layer 3A pyramidal per MC	$n_{MC}^{PYR-L3A}$	5	LTM intra-HC–inter-MC weight	$w_{InterMC}^{IntraHC}$	−4.82 $w_{gain}^{syn}$
Layer 3B pyramidal per MC	$n_{MC}^{PYR-L3B}$	5			
Layer 4 pyramidal per MC	$n_{MC}^{PYR-L4}$	30	LTM inter-HC–coactive MC weight	$w_{CoactiveMC}^{InterHC}$	3.08 $w_{gain}^{syn}$
Basket cells per MC	$n_{MC}^{basket}$	4	LTM inter-HC–competing MC weight	$w_{Competin\;gMC}^{Inter\;HC}$	−4.28 $w_{gain}^{syn}$

Layer 4 not simulated in LTM.

**Table 3:** Projections

Scope	Source	Target	Type	Symbol	Value
Cortical area	Pyramidal	Basket	Probability	*p_P-B_*	0.7
	Pyramidal	Basket	Conductance (static)	*g_P-B_*	+3.5 nS
	Basket	Pyramidal	Probability	*p_B-P_*	0.7
	Basket	Pyramidal	Conductance (static)	*g_B-P_*	−20 nS
	L23e	L23e	Probability	*p_L_*_23_*_e_*_-_*_L_*_23_*_e_*	0.2
	L23e	L23e	AMPA gain (BCPNN)	$w_{gain}^{AMPA}$	3.93nS
	L23e	L23e	NMDA gain (BCPNN)	$w_{gain}^{NMDA}$	0.21nS
	L4e	L23e	Probability	*p_L_*_4_*_e_*_-_*_L_*_23_*_e_*	0.25
	L4e	L23e	Conductance (static)	*g_L_*_4_*_e_*_-_*_L_*_23_*_e_*	25 nS
Feed forward	LTM L3Ae	STM MC	Probability	$p_{L3Ae-MC}^{FF}$	0.0015
	LTM L3Ae	STM MC	Branching factor	$b_{L3Ae-MC}^{FF}$	0.25
	LTM L3Ae	STM L23e	Conductance (static)	$g_{L3Ae-L23e}^{FF}$	±7.2 nS
	LTM L3Ae	STM L4e	Conductance (static)	$g_{L3Ae-L4e}^{FF}$	±7.2 nS
Feedback	STM PYR	LTM PYR	Probability	$p_{P-P}^{FB}$	0.0066
	STM L3Be	LTM HC	Branching factor	$b_{L3Be-HC}^{FB}$	0.25
	STM L3Be	LTM L23e	AMPA gain (BCPNN)	$w_{FB}^{AMPA}$	7.07 nS
	STM L3Be	LTM L23e	NMDA gain (BCPNN)	$w_{FB}^{NMDA}$	0.4 nS

Code for the NEST implementation of the BCPNN synapse is openly available (see Code accessibility).

### Axonal conduction delays

We compute axonal delays *t_ij_* between presynaptic neuron *i* and postsynaptic neuron *j*, based on a constant conduction velocity *V* and the Euclidean distance between respective columns. Conduction delays were randomly drawn from a normal distribution with mean according to the connection distance divided by conduction speed and with a relative SD of 15% of the mean in order to account for individual arborization differences and varying conduction speeds as a result of axonal thickness/myelination. Further, we add a minimal conduction delay $t_{min}^{syn}$ of 1.5 ms to reflect not directly modeled delays, such as diffusion of transmitter over the synaptic cleft, dendritic branching, thickness of the cortical sheet, and the spatial extent of columns, as follows:

(10) $$\begin{array}{ll} \bar{t_{ij}}=\frac{\sqrt{{\left(x_{i}-x_{j}\right)}^{2}+{\left(y_{i}-y_{j}\right)}^{2}}}{V}+t_{mn}^{syn}ms & t_{ij}∼N(\bar{t_{ij}},.15\bar{t_{ij}}). \end{array}$$

### STM network architecture

The model organizes cells in the three simulated cortical areas into grids of nested hypercolumns (HCs) and MCs, sometimes referred to as macro columns, and “functional columns,” respectively. The STM network is simulated with $n_{HC}^{STM}=25$ HCs spread out on a grid with spatial extent of 17 × 17 mm. This spatially distributed network of columns has sizable conduction delays due to the distance between columns and can be interpreted as a spatially distributed subsampling of columns from the extent of dorsolateral PFC (e.g., BA 46 and 9/46, which also have a combined spatial extent of ∼289 mm^2^ in macaque).

Each of the nonoverlapping HCs has a diameter of ∼640 μm, comparable to estimates of cortical column size (Mountcastle, 1997), contains 48 basket cells, and its pyramidal cell population has been divided into 12 MCs. This constitutes another subsampling from the ∼100 MCs per HC when mapping the model to biological cortex. We simulate 20 pyramidal neurons per MC to represent approximately the layer 2 population of an MC, 5 cells for layer 3A, 5 cells for layer 3B, and another 30 pyramidal cells for layer 4, as macaque BA 46 and 9/46 have a well developed granular layer (Petrides and Pandya, 1999). The STM model thus contains ∼18,000 simulated pyramidal cells in four layers (although layers 2, 3A, and 3B are often treated as one layer 2/3).

### STM network connectivity

The most relevant connectivity parameters are found in Tables 1, 2, and 3. Pyramidal cells project laterally to basket cells within their own HC via AMPA-mediated excitatory projections with a connection probability of *p_p_*_–_*_B_* (i.e., connections are randomly drawn without duplicates until the target fraction of all possible pre–post connections exist). In turn, they receive GABAergic feedback (FB) inhibition from basket cells (*p_B_*_–_*_p_*) that connect via static inhibitory synapses rather than plastic BCPNN synapses. This strong loop implements a competitive soft WTA (winner-take-all) subnetwork within each HC (Douglas and Martin, 2004). Local basket cells fire in rapid bursts, and induce alpha/beta oscillations in the absence of attractor activity and gamma, when attractors are present and active.

Pyramidal cells in layer 2/3 form connections both within and across HCs at connection probability *p_L_*_23_*_e_*_-_*_L_*_23_*_e_*. These projections are implemented with plastic synapses and contain both AMPA and NMDA components, as explained in the subsection Spike-based BCPNN learning rule. Connections across columns and areas may feature sizable conduction delays due to the implied spatial distance between them (Table 1).

Pyramidal cells in layer 4 project to pyramidal cells of layer 2/3, targeting 25% of cells within their respective MC only. Experimental characterization of excitatory connections from layer 4 to layer 2/3 pyramidal cells have confirmed similarly high fine-scale specificity in rodent cortex (Yoshimura and Callaway, 2005) and, in turn, full-scale cortical simulation models without functional columns have found it necessary to specifically strengthen these connections to achieve defensible firing rates (Potjans and Diesmann, 2014).

In summary, the STM model thus features a total of 16.2 million plastic AMPA- and NMDA-mediated connections between its 18,000 simulated pyramidal cells, as well as 67,500 static connections from 9000 layer four pyramidals to layer 2/3 targets within their respective MC, and 1.2 million static connections to and from 1200 simulated basket cells.

### LTM network

**Figure 2.**
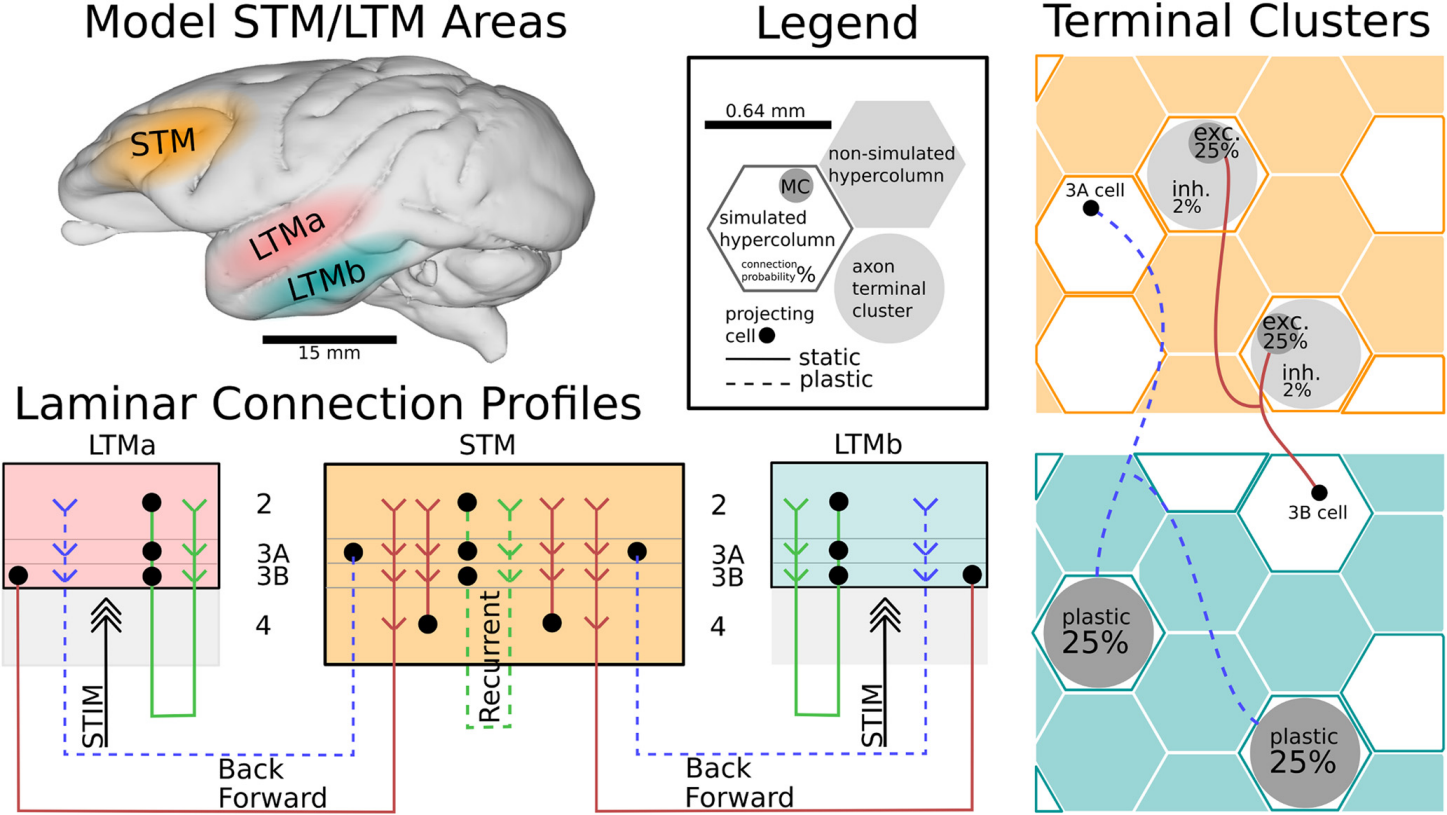
Schematic of modeled connectivity within and across representative STM and LTM areas in macaque. STM features 25 HCs, whereas LTMa and LTMb both contain 16 simulated HCs. Each network spans several hundred square millimeters, and the simulated columns constitute a spatially distributed subsample of biological cortex, defined by conduction delays. Pyramidal cells in the simulated supragranular layers form connections both within and across columns. STM features an input layer 4 that shapes the input response of cortical columns, whereas LTM is instead stimulated directly to cue the activation of previously learned long-term memories. Additional corticocortical connections (feedforward in brown, feedback in dashed blue) are sparse (<1% connection probability) and implemented with terminal clusters (rightmost panels) and specific laminar connection profiles (bottom left). The connection schematic illustrates laminar connections realizing a direct supragranular forward-projection, as well as a common supragranular backprojection. Layer 2/3 recurrent connections in STM (dashed green) and corticocortical backprojections (dashed blue) feature fast Hebbian plasticity. For an in-depth model description, including the columnar microcircuits, please refer to Materials and Methods and Figure 1.

We simulate two structurally identical LTM networks, referred to as LTMa and LTMb. LTM networks may be interpreted as a spatially distributed subsampling of columns from areas of the parietotemporal cortex commonly associated with modal LTM stores. For example, inferior temporal cortex (ITC) is often referred to as the storehouse of visual LTM (Miyashita, 1993). Two such LTM areas are indicated in Figure 2.

We simulate $n_{HC}^{LTM}=16$ HCs in each area and 9 MCs per HC (Tables 1, 2, 3, for further details). Both LTM networks are structurally very similar to the previously described STM, yet they do not feature plasticity among their own cells, beyond short-term dynamics in the form of synaptic depression. Unlike STM, LTM areas also do not feature an input layer 4, but are instead stimulated directly to cue the activation of previously learned long-term memories (see Stimulation protocol). Various previous models with identical architecture have demonstrated how attractors can be learned via plastic BCPNN synapses (Lansner et al., 2013; Tully et al., 2014, 2016; Fiebig and Lansner, 2017). We load each LTM network with nine orthogonal attractors [see Fig. 4*B*, 10 in the example (which features two sets of five memories each)]. Each memory pattern consists of 16 active MCs, distributed across the 16 HCs of the network. We load in BCPNN weights from a previously trained network (Table 2), but thereafter set *κ* = 0 to deactivate plasticity of recurrent connections in LTM stores.

In summary, the two LTM models thus feature a total of 7.46 million connections between 8640 pyramidal cells, as well as 435,456 static connections to and from 1152 basket cells.

### Interarea connectivity

In this model, we focus on layers 2/3, as its high degree of recurrent connectivity (Thomson et al., 2002; Yoshimura and Callaway, 2005) supports attractor function. The high fine-scale specificity of dense stellate cell (Yoshimura et al., 2005) and double-bouquet cell inputs (DeFelipe et al., 2006; Chrysanthidis et al., 2019) enable strongly coding subpopulations in the superior layers of functional columns. This fits with the general observation that layers 2/3 are more input selective than the lower layers (Crochet and Petersen, 2009; Sakata and Harris, 2009) and thus of more immediate concern to our computational model.

The recent characterization of supragranular feedforward (FF) and FB projections (from large cells in layer 3B and 3A, respectively), between association cortices and at short and medium cortical distances (Markov et al., 2014), allows for the construction of a basic cortical hierarchy without explicit representation of infragranular layers (and its long-range FB projections from large cells in layer 5 and 6). This is not to say that nothing would be gained by explicitly modeling infragranular layers, but it would go beyond the scope of this model.

Accordingly, our model implements supragranular FF and FB pathways between cortical areas that are at a medium distance in the cortical hierarchy. The approximate cortical distance between ITC and dlPFC in macaque is ∼40 mm and with an axonal conductance speed of 2 m/s, distributed conduction delays in our model (Eq. 10) average just >20 ms between these areas (Girard et al., 2001; Thorpe and Fabre-Thorpe, 2001; Caminiti et al., 2013).

In the forward path, layer 3B cells in LTM project toward STM (Fig. 2). We do not draw these connections one by one, but as branching axons targeting 25% of the pyramidal cells in a randomly chosen MC (the chance of any layer 3B cell to target any MC in STM is only 0.15%). The resulting split between targets in layer 2/3 and 4 is typical for FF connections at medium distances in the cortical hierarchy (Markov et al., 2014) and has important functional implications for the model (LTM-to-STM forward dynamics). We also branch off some inhibitory corticocortical connections as follows: for every excitatory connection within the selected targeted MC, an inhibitory connection is created from the same pyramidal layer 3B source cell onto a randomly selected cell outside the targeted MC, but inside the local HC. This way of drawing random forward-projections retains a degree of functional specificity due to its spatial clustering and yields patchy sparse forward-projections as observed in the cortex (Houzel et al., 1994; Voges et al., 2010), with a resulting interarea connection probability of only 0.0125% (648 axonal projections from L3B cells to STM layers 2/3 and 4 results in ∼20,000 total connections after branching, as described above.

In the FB path, we draw sparse plastic connections from layer 3A cells in STM to layer 2/3 cells in LTM: branching axons target 25% of the pyramidal cells in a randomly chosen HC in LTM, simulating a degree of axonal branching found in the literature (Zufferey et al., 1999). Using this method, we obtain biologically plausible sparse and structured FB projections with an interarea connection probability of 0.66%, which, unlike the forward pathway, do not have any built-in MC specificity but may develop such through activity-dependent plasticity. More parameters on corticocortical projections can be found in Table 3. On average, each LTM pyramidal cell receives ∼120 corticocortical connections from STM. Because ∼5% of STM cells fire together during memory reactivation (see Results), this means that a mere 6 active synapses per target cell are sufficient for driving (and thus maintaining) LTM activity from STM (there are 96 active synapses from coactive pyramidal cells in LTM).

Notably LTMa and LTMb have no direct pathways connecting them in our model since we assume that the use of previously not associated stimuli in our simulated multimodal tasks and further, that plasticity of biological connections between them are likely too slow (LTP timescale) to make a difference in WM dynamics. This arrangement also guarantees that any binding of long-term memories across LTM areas must be the result of interaction via STM instead. Overall in our model, corticocortical connectivity is very sparse, <1% on a cell-to-cell basis.

### Stimulation protocol

The term *I*_ext_ in Equation 1 subsumes specific and unspecific external inputs. To simulate unspecific input from nonsimulated columns, and other areas, pyramidal cells are continually stimulated with a zero mean noise background throughout the simulation. In each layer, two independent Poisson sources generate spikes at rate $r_{bg}^{layer}$ and connect onto all pyramidal neurons in that layer, via nondepressing conductances $\pm g_{bg}$ (Table 2). Before each simulation, we distribute the initial values of all plastic weights by a process of learning from 1.5 s low, unstructured background activity (Table 2; $r_{bg-low}^{L23}$). To cue the activation of a specific memory pattern (i.e., attractor), we excite LTM pyramidal cells belonging to a memory patterns component MC with an additional excitatory Poisson spike train (rate, *r*_cue_, length, *t*_cue_; conductance, *g*_cue_). As LTM patterns are strongly encoded in each LTM, a brief 50 ms stimulus is usually sufficient to activate any given memory.

### Synthetic field potentials and spectral analysis

We estimate local field potentials (LFPs) by calculating a temporal derivative of the average low-pass filtered (cutoff frequency at 250 Hz) potential for all pyramidal cells in local populations at every time step, similarly to the approach adopted by Ursino and La Cara (2006). Although LFP is more directly linked to the synaptic activity (Logothetis, 2003), the averaged membrane potentials have been reported to be correlated with LFPs (Okun et al., 2010). In particular, low pass-filtered components of synaptic currents reflected in differentiated membrane potentials appear to carry the portion of the power spectral content of extracellular potentials that is relevant to our key findings (Lindén et al., 2010). As regards the phase response of estimated extracellular potentials, the delays of different frequency components are spatially dependent (Lindén et al., 2010). However, irrespective of the LFP synthesis, the phase-related phenomena reported in this study remain qualitatively unaffected since they hinge on relative rather than absolute phase values.

**Figure 3.**
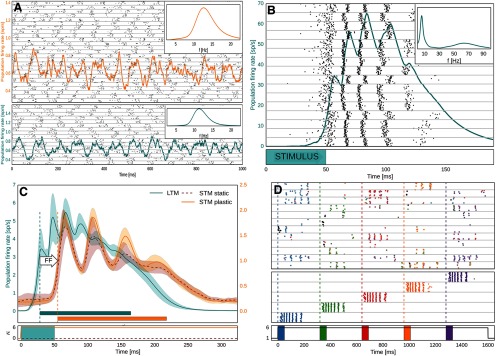
Basic network behavior in spike rasters and population firing rates. Activity in the untrained network under strong background input. ***A***, Subsampled spike raster of STM (top) and LTM (bottom) layer 2/3 activity. HCs are separated by gray horizontal lines. Global oscillations in the alpha range (10–13 Hz) characterize this activity state in both STM (top) and LTM (bottom) in the absence of attractors. Inset, Power spectral density of LFP of each network. ***B***, Cued LTM memory activation express as fast oscillation bursts of selective cells (50–80 Hz), organized into a theta-like envelope (4–8 Hz), see also power spectrum inset. The gamma band is broad due to the varying lengths of the underlying cycles (i.e., noticeably increasing over the short memory activation period). The underlying spike raster shows layer 2/3 activity of the activated MC in each HC, revealing spatial synchronization. The brief stimulus is a memory-specific cue. ***C***, LTM-to-STM forward dynamics as shown in population firing rates of STM and LTM activity following LTM activation induced by a 50 ms targeted stimulus at time 0. LTM-driven activations of STM are characterized by an FF delay. Shadows indicate the SD of 100 peristimulus activations in LTM (blue) and STM (orange) with and without plasticity enabled (dashed, dark orange). Horizontal bars indicate the activation half-width (Materials and Methods). Onset is denoted by vertical dashed lines. The stimulation of LTM and the activation of plasticity is denoted underneath. ***D***, Subsampled spike raster of STM (top) and LTM (middle) during forward activation of the untrained STM by five different LTM memory patterns, triggered via specific memory cues in LTM at times marked by the vertical dashed lines. Bottom spike raster shows LTM layer 2/3 activity of one selective MC per activated pattern (colors indicate different patterns). Top spike raster shows layer 2/3 activity of one HC in STM. STM spikes are colored according to each cells dominant pattern selectivity (based on the memory pattern correlation of individual STM cell spiking during initial pattern activation, see Materials and Methods, Spike train analysis and memory activity tracking). Bottom, The five stimuli to LTM (colored boxes) and modulation of STM plasticity (black line). Extended Data Figure 3-1 shows basic network behavior in spike rasters and population firing rates under low-input feature fluctuations in membrane voltages and low-rate, asynchronous spiking activity, while Extended Data Figure 3-2 shows network activity during plasticity-modulated stimulation with 20% spatial extent, illustrating the impact of conductance delays on cortical dynamics (see Model robustness).

Most spectral analyses have been conducted on the synthesized field potentials with the exception of population firing rates, shown in Figure 3, *A* and *B*. Spectral information is extracted with a multitaper approach using a family of orthogonal tapers produced by Slepian functions (Slepian, 1978; Thomson, 1982), with frequency-dependent window lengths corresponding to five to eight oscillatory cycles and frequency smoothing corresponding to 0.3–0.4 of the central frequency, which was sampled with the resolution of 1 Hz (this configuration implies that two to three tapers are usually used). To obtain the spectral density, spectrotemporal content is averaged within a specific time interval.

10.1523/ENEURO.0374-19.2020.f3-1 Figure 3-1 Basic network behavior in spike rasters and population firing rates under low input. The untrained networks STM (top) and LTM (bottom) feature low-rate, asynchronous activity (CV2 = 0.7 ± 0.2). The underlying spike raster shows layer 2/3 activity in each HC (separated by gray horizontal lines) in the simulated network. Download Figure 3-1, TIF file.

10.1523/ENEURO.0374-19.2020.f3-2 Figure 3-2 Network activity during plasticity-modulated stimulation with 20% spatial extent. Subsampled spike raster of the layer 2/3 population in a hypercolumn of STM (top), and five coding minicolumns in LTMa (second row) and LTMb (third row), respectively, during plasticity-modulated stimulation (i.e., encoding) of five paired LTM patterns. Without sufficient conduction delays, memory activations collapse into very brief bursts (with the exception of the last pattern here), and STM cannot effectively activate from, or subsequently encode, such brief activations (Fig. 3*B*,*D*). Download Figure 3-2, TIF file.

The coherence for a pair of synthesized field potentials at the spatial resolution corresponding to a hypercolumn was calculated using the multitaper auto-spectral and cross-spectral estimates. The complex value of coherence (Carter, 1987) was evaluated first based on the spectral components averaged within 0.5 s windows. Next, its magnitude was extracted to produce the time-windowed estimate of the coherence amplitude. In addition, phase-locking statistics were estimated to examine synchrony without the interference of amplitude correlations (Lachaux et al., 1999; Palva et al., 2005). In particular, the phase-locking value (PLV) between two signals with instantaneous phases *Φ*_1_(*t*) and *Φ*_2_(*t*) was evaluated within a time window of size *N *=* *0.5 s as follows:

$$\text{PLV}=\frac{1}{N}\left|\sum_{i=1}^{N}\exp\left(j\left(\Phi_{1}\left(t_{i}\right)-\Phi_{2}\left(t_{i}\right)\right)\right)\right|.$$

The instantaneous phase of the signals was estimated from their analytic signal representation obtained using a Hilbert transform. Before the transform was applied, the signals were narrow band filtered with low-time domain spread, finite-impulse response filters (in the forward and reverse directions to avoid any phase distortions). The analysis was performed mainly for gamma-range oscillations. A continuous PLV estimate was obtained with a sliding window approach, and the average along with SE were calculated typically over 25 trials.

### Spike train analysis and memory activity tracking

We track memory activity in time by analyzing the population firing rate of pattern-specific and network-wide spiking activity usually using an exponential moving average filter time constant of 20 ms. We do not use an otherwise common low-pass filter with symmetrical window, because we are particularly interested in characterizing activation onsets and onset delays. As activations are characterized by sizable gamma-like bursts, a simple threshold detector can extract candidate activation events and decode the activated memory. This is trivial in LTM due to the known nature of its patterns. In STM, we decode the stimulus specificity of each cell individually by finding the maximum correlation between input pattern and the untrained STM spiking response in the 320 ms following cue onset (which is the stimulation interval during the plasticity-modulated stimulation period; Fig. 3*D*) following the pattern cue to LTM. Thereafter, we can filter the population response of cells in STM with the same selectivity on that basis to obtain a more robust readout. We validate the specificity by means of cross-correlations, which reveal that the pattern-specific populations are rather orthogonal according to the covariance matrix (off-diagonal magnitude, <0.1). In all three networks, we measure the onset and offset of pattern activity by thresholding each individual activation at half of its population peak firing rate. In LTM, we further check pattern completion by analyzing component MC activation. Whenever targeted stimuli are used, we analyze peristimulus activation traces. When activation onsets are less predictable, such as during free STM-paced maintenance, we extract activation candidates via a threshold detector trained at the 50th percentile of the cumulative distribution of the population firing rate signal.

### Code accessibility

We used the NEST simulator (Gewaltig and Diesmann, 2007) version 2.2 for our simulations (RRID:SCR_002963), running on a Cray XC-40 Supercomputer of the PDC Centre for High Performance Computing. The custom-built spiking neural network implementation of the BCPNN learning rule for MPI (message passing interface) parallelized NEST is freely available on github (https://github.com/Florian-Fiebig/BCPNN-for-NEST222-MPI) and is included in the Extended Data 1. Further, the model is also available on ModelDB (https://modeldb.yale.edu/257610).

### Model robustness

Our model incorporates a plethora of biological constraints, such as estimates of the extent and distance of areas (e.g., STM patch size approximates macaque dlPFC and is 40 mm from either LTM patch), laminar cell distributions ($n_{MC}^{pYR-L2}$, $n_{MC}^{pYR-L3b}$,…), and hypercolumnar size. The model also abides by various electrophysiological constraints, such as plausible EPSP, IPSP sizes, estimates on laminar connection densities, laminar characterization of cortical FF/FB pathways with remote patchy connectivity, estimates on axonal conductance speeds, dendritic arbor sizes (branching factors), commonly accepted synaptic time constants for various receptor types, depression, adaptation, and builds on top of established models, such as the neuron model or the synaptic resource model. References to many of these constraints can be found throughout the Materials and Methods.

Because our model is quite complex and synthesizes many different components and processes, it is beyond the scope of this work to perform a detailed parameter sensitivity analysis. However, from our extensive simulations we conclude that it is robust and degrades gracefully. Almost all uncertain parameters can be varied ±30% without breaking WM function. The model is dramatically subsampled, and scaling up would be possible. This could be expected to further improve overall robustness. Highly related modular cortical network models have been studied extensively previously (Lundqvist et al., 2010, 2011; Tully et al., 2013, 2014; Fiebig and Lansner, 2017). For example, the model sensitivity to important short-term plasticity parameters affecting active maintenance mechanisms and intermittent gamma bursts (e.g., neural adaptation and synaptic depression time constants) were specifically explored in a single-network model (Fiebig and Lansner, 2017; see Fig. 8).

In the following, we briefly address new aspects of model sensitivity, previously unexplored, such as the parameterization of corticocortical connectivity, spatial scale (and associated conduction delays), as well as the transient modulation of Hebbian plasticity during rapid WM encoding.

In the FB pathway, a mere 0.6% connectivity is sufficient to support LTM activation in maintenance and recall. As rigorous testing (data not shown here) revealed, lower connectivity degrades WM capacity, unless we increase the total number of coactive STM cells by other means. FF connectivity can be even lower (0.015% in this model) because terminal clusters in STM are smaller and provide more information contrast (corticocortical connectivity). In both cases, our model uses very sparse connectivity, yet it could be increased or decreased if single synaptic currents were reduced/increased, respectively. Somewhat peculiarly, we also found that we needed to increase the corticocortical conductance of the backprojections ($w_{FB}^{syn}$) by the same factor of 1.8 (over the local conductance gain $w_{gain}^{syn}$) as another highly detailed multiarea model of macaque visual cortex (Schmidt et al., 2018) to achieve functional WM at the stated long-distance connection probabilities.

There are upper and lower limits on conduction delays in our model. When corticocortical conduction delays exceed 65 ms (corresponding to 130 mm in distance), STM FB can no longer activate the LTM network because bursts desynchronize before they arrive. STM and LTM could be adjacent, as we briefly mention at the end of the Results section, but there is a minimum spatial scale for each component network. The length of gamma bursts decreases if we reduce the spatial extent (and thus the connection delays between HCs) by 45%. At 20%, when the largest inter-HC delays fall to <5 ms (Extended Data Fig. 3–2), the spiking activity of activated memories collapses into a single brief burst, which degrades learning and effective information transmission both within and across networks. Networks may be much smaller, however, if this is compensated by slower axonal conductance velocities (<2 mm/ms). Furthermore, we verified that the relative temporal delay dither in Equation 10 can be varied considerably (0–30%) without noticeable effects on memory performance.

The Hebbian plasticity of the model can be modulated via the parameter *κ* (Eq. 7). While *κ* is normally 1 (*κ*_normal_, a transient increase of *κ* = *κ*_encoding_; Table 1), it enables rapid, one-shot encoding in STM (Fig. 3*D*). Halving or doubling *κ*_encoding_ affects the overall working memory performance of the model only slightly, as measured by the number of items maintained during the delay period, or the overall rate of gamma bursts (Extended Data Fig. 4–4). It is, however, not possible to maintain normal WM operation without upregulating plasticity during encoding (leaving *κ*_encoding_ = *κ*_normal_), unless additional compensatory changes are made to increase STM excitability, background excitation, or excitatory long-range connectivity. The strong correlation between working memory load and gamma-burst rate was previously discussed by Fiebig and Lansner (2017) in the context of evidence from multi-item WM recordings in macaque by Lundqvist et al. (2016).

## Results

Our model implements WM function arising from the interactions of STM and LTM networks, which manifest as multi-modal memory binding phenomena. To this end, we simulate three cortical patches with significant biophysical detail: one STM and two LTM networks (LTMa, LTMb), representing PFC and parietotemporal areas, respectively (Fig. 2). The computational network model used here represents a detailed modular cortical microcircuit architecture in line with previous models (Lundqvist et al., 2006, 2011; Tully et al., 2016). Like those models, the new model can reproduce a wide range of mesoscopic and macroscopic biological manifestations of cortical memory function, including complex oscillatory dynamics and synchronization effects (Silverstein and Lansner, 2011; Lundqvist et al., 2011, 2013). The current model is built directly on a recent STM model of human word-list learning (Fiebig and Lansner, 2017). We subdivided the associative cortical layer 2/3 network of that model into layers 2, 3A, and 3B. Importantly, we also extended this model with an input layer 4 and corticocortical connectivity to LTM stores in temporal cortical regions. This large, multiarea network model synthesizes many different anatomic and electrophysiological cortical data and produces complex output dynamics. Here, we specifically focus on the dynamics of memory specific subpopulations in the interaction of STM and LTM networks.

We introduce the operation of the WM model in several steps. First, we take a brief look at background activity and active memory states in isolated cortical networks of this kind to familiarize the reader with some of its dynamical properties. Second, we describe the effect of memory activation on STM with and without plasticity. Third, we add the plastic backprojections from STM to LTM and monitor the encoding and maintenance of several memories in the resulting STM–LTM loop. We track the evolution of acquired cell assemblies with shared pattern selectivity in STM and show their important role in WM maintenance (called delay activity). We then demonstrate that the emerging WM network system is capable of flexibly updating the set of maintained memories. Finally, we simulate multimodal association and analyze its dynamical correlates. We explore temporal characteristics of network activations, the accompanying oscillatory behavior of the synthesized field potentials, cross-cortical delays as well as gamma-band coupling (coherence and phase synchronization) between LTM networks during WM encoding, maintenance, and cue-driven associative recall of multimodal memories (LTMa–LTMb pairs of associated memories).

### Background activity and activated memory

At sufficiently high background input levels, the empty network transitions from asynchronous spiking activity into a state characterized by global oscillations of the population firing rates in the alpha/beta range (Fig. 3*A*). This is largely an effect of fast feedback inhibition from local basket cells (Fig. 1), high connection density within MCs, and low latency local spike transmission (Lundqvist et al., 2010). If the network has been trained with structured input so as to encode memory (i.e., attractor states), background noise, or a specific cue (Materials and Methods) can trigger memory item reactivations accompanied by fast broadband oscillations modulated by an underlying slow oscillation in the lower theta range (∼4–8 Hz; Lundqvist et al., 2011; Herman et al., 2013; Fig. 3*B*). The spiking activity of memory activations (called attractors) is short lived due to neural adaptation and synaptic depression. When unspecific background excitation is very strong, this can result in a random walk across stored memories (Lundqvist et al., 2011; Fiebig and Lansner, 2017).

### LTM-to-STM forward dynamics

We now consider cued activation of several memories embedded in LTM. Each HC in LTM features selectively coding MCs for given memory patterns that activate synchronously in theta-like cycles each containing several fast oscillation bursts (Fig. 3*B*). Five different LTM memory patterns are triggered by brief cues, accompanied by an upregulation of STM plasticity (Fig. 3*D*, bottom). To indicate the spatiotemporal structure of evoked activations in STM, we also show a simultaneous subsampled STM spike raster (Fig. 3*D*, top). STM activations are sparse (∼5%), but despite this, nearby cells (in the same MC) often fire together. The distributed, patchy character of the STM response to memory activations (Fig. 3*D*, top) is shaped by branching forward-projections from LTM layer 3B cells, which tend to activate cells that are close by. STM input layer four receives half of these corticocortical connections and features very high fine-scale specificity in its projections to layer 2/3 pyramidal neurons, which furthers the recruitment of local clusters with shared selectivity. STM cells initially fire less than those in LTM because the latter received a brief, but strong, activation cue and have strong recurrent connections if they code for the same embedded memory pattern. STM spikes in Figure 3*D* are colored according to the dominant memory pattern selectivity of the cells (Materials and Methods, Spike train analysis and memory activity tracking), which reveals that STM activations are mostly nonoverlapping as well. Unlike the organization of LTM with strictly nonoverlapping memory patterns, MC activity in STM is not exclusive to any given input pattern. Nevertheless, nearby STM cells often develop similar pattern selectivity. On the other hand, different stimulus patterns typically develop quite nonoverlapping STM representations. This is due to the randomness in LTM–STM connectivity, competition via basket cell feedback inhibition, and short-term dynamics, such as neural adaptation and synaptic depression. STM neurons that have recently been activated by a strong, bursting input from LTM are refractory and thus less prone to spike again for some time thereafter (*τ*_rec_ and $\tau_{I_{w}}$; Table 1), further reducing the likelihood of creating overlapping STM representations for different patterns.

**Figure 4.**
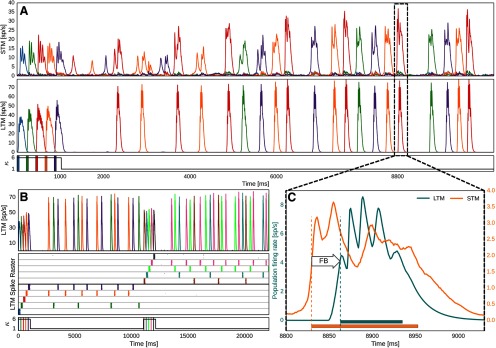
Encoding and feedback-driven reactivation of LTM. ***A***, Firing rates of pattern-specific subpopulations in STM and LTM during encoding and subsequent maintenance of five memories. Just as in the plasticity-modulated stimulation phase shown in Figure 2*D*, five LTM memories are cued via targeted 50 ms stimuli (shown underneath). Plasticity of STM and its backprojections is again elevated sixfold during the initial memory activation. Thereafter, a strong noise drive to STM causes spontaneous activations and plasticity induced consolidation of pattern-specific subpopulations in STM (lower plasticity, *κ* = 1). Backprojections from STM cell assemblies help reactivate associated LTM memories. ***B***, Updating of WM. Rapid encoding and subsequent maintenance of a second group of memories following an earlier set. The LTM spike raster shows layer 2/3 activity of one LTM HC (MCs separated by gray horizontal lines), and the population firing rate of pattern-specific subpopulations across the whole LTM network is seen above. Underneath, we denote stimuli to LTM and the modulation of plasticity, *κ*, in STM and its backprojections. ***C***, STM-to-LTM loop dynamics during a spontaneous reactivation event. STM-triggered activations of LTM memories are characterized by a feedback delay and a second peak in STM after LTM activations. Horizontal bars at the bottom indicate activation half-width (Materials and Methods). Onset is denoted by vertical dashed lines. Extended Data Figure 4-1 shows a more detailed spike raster of WM encoding and maintenance. Extended Data Figures 4-2 and 4-3 show spike rates and a subsampled spike rasters during WM updating and maintenance. Extended Data Figure 4-4 shows the sensitivity of delay activity to the plasticity modulation *κ_v_* during encoding.

Figure 3*C* shows peristimulus population firing rates of both STM and LTM networks (the mean across 100 trials with five triggered memories each). There is a bottom-up response delay between stimulus onset at *t* = 0 and LTM activation, as well as a substantial forward delay. Oscillatory activity in STM is lower than in LTM mostly because the untrained STM lacks strong recurrent connections. It is thus less excitable, and therefore does not trigger its basket cells (the main drivers of fast oscillations in our model) as quickly as in LTM. Fast oscillations in STM and the amplitude of their theta-like envelope build up within a few seconds as new cell assemblies become stronger [Fig. 4*A* (see also Fig. 8)]. As seen in Figure 3*B*, bursts of coactivated MCs in LTM can become asynchronous during activation. Dispersed forward axonal conduction delays further decorrelate this gamma-like input to STM. Activating strong plasticity in STM (*κ* = κ_p_; Materials and Methods; Table 1) has a noticeable effect on the amplitude of stimulus-locked oscillatory STM activity after as little as 100 ms (Fig. 3*C*, STM).

10.1523/ENEURO.0374-19.2020.f4-1 Figure 4-1 Encoding and feedback-driven reactivation of long-term memories. Subsampled spike raster of STM (top) and LTM (bottom) during encoding and subsequent maintenance of five memories (the first pattern is not maintained in this simulation). During the initial plasticity-modulated stimulation phase, five LTM memories are cued via targeted 50 ms stimuli (shown underneath). Plasticity of STM and its backprojections is modulated during this initial memory activation (Fig. 3*D*). Thereafter, a strong noise drive to STM causes spontaneous activations and plasticity-induced consolidation of pattern-specific subpopulations in STM. Backprojections reactivate associated LTM memories. Top, STM spike raster shows layer 2/3 activity in a single HC. MCs are separated by gray horizontal lines. STM spikes are colored according to each cell’s dominant LTM pattern-correlation, similar to Figure 2*D*. Bottom, LTM spike raster only shows the activity of five coding MC in a single LTM HC, but indicates the activation of distributed LTM memory patterns. LTM spikes are colored according to the pattern specificity of each cell. Download Figure 4-1, TIF file.

10.1523/ENEURO.0374-19.2020.f4-2 Figure 4-2 Spike rates during WM updating. Population firing rates of pattern-specific subpopulations in STM and LTM during encoding and subsequent maintenance of two sets of five LTM memories. After encoding and 10 s maintenance of the first set, WM contents are overwritten with the second set of memories, maintained thereafter in spontaneous reactivation events. Bottom: Stimuli to LTM and modulation of plasticity. Download Figure 4-2, TIF file.

10.1523/ENEURO.0374-19.2020.f4-3 Figure 4-3 Spike raster during WM updating. Subsampled spike raster of the layer 2/3 population in a hypercolumn of STM (top) and LTM (bottom) respectively during encoding and subsequent maintenance of two sets of five LTM memories. STM spikes are colored according to each cells dominant pattern-selectivity. LTM spikes are colored according to the pattern-specificity of each cell. After encoding and 10 s maintenance of the first set, WM contents are overwritten with the second set of memories, maintained thereafter. Plasticity is temporarily boosted during the initial activation of LTM attractors (see preceding figure). Strong noise drive to STM causes spontaneous reactivations and consolidation of pattern-specific subpopulations in STM following each stimulation period. Download Figure 4-3, TIF file.

10.1523/ENEURO.0374-19.2020.f4-4 Figure 4-4 Sensitivity of WM delay activity to the plasticity modulation *κ*_encoding_ during encoding. The size of the set of actively maintained items (top) and the average rate of gamma burst events (bottom) over the 10 s delay period is reasonably stable around the operating point of *κ*_encoding_ = 6. Results are averaged from 84 simulation runs with varying amounts of plasticity modulation. Error bars denote the SE. Download Figure 4-4, TIF file.

### Multi-item working memory

In Figure 3*D*, we have shown pattern-specific subpopulations in STM emerging from FF input. Modulated STM plasticity allows for the quick formation of rather weak STM cell assemblies from one-shot learning. When we include plastic STM backprojections, these assemblies can serve as an index for specific LTM memories and provide top-down control signals for memory maintenance and retrieval. STM backprojections with fast Hebbian plasticity can index multiple activated memories in the closed STM–LTM loop. In Figure 4*A*, we show network activity following targeted activation of five LTM memories. Under an increased unspecific noise drive ($r_{bg-high}^{L23}$; Table 2), STM cell assemblies formed during the brief plasticity-modulated stimulus phase (Fig. 3*D*) may activate spontaneously. These brief bursts of activity are initially weak and different from the theta-like cycles of repeated fast bursting seen in LTM attractor activity.

STM recurrent connections remain plastic (*κ* = 1) throughout the simulation, so each reactivation event further strengthens memory-specific cell assemblies in STM. As a result, there is a noticeable ramp-up in the strength of STM pattern-specific activity over the course of the delay period (Fig. 4*A*, increasing burst length and amplitude). STM backprojections are also plastic and thus acquire memory specificity from STM–LTM coactivations, especially during the initial stimulation phase. Given enough STM cell assembly firing, their sparse but potentiated backprojections can trigger associated memories in LTM. Weakly active assemblies may fail to do so. In the example of Figure 4*A*, we can see a few early STM reactivations that are not accompanied (or quickly followed) by a corresponding LTM pattern activation (of the same color) in the first 2 s after the plasticity-modulated stimulation phase. When LTM is triggered, there is a noticeable FB delay (Fig. 4*C*), which we will address together with aforementioned FF delays in the analysis of recall dynamics during a multi-item, multimodal recall task.

Cortical FF and FB pathways between LTM and STM form a loop, so each LTM activation will again feed into STM, typically causing a second peak of activation in STM 40 ms after the first (Fig. 4*C*). The forward delay from LTM to STM, which we have seen earlier in the stimulus-driven input phase (Fig. 3*C*), is still evident here in this delayed secondary increase of the STM activation following LTM onset. The reverberating cross-cortical activation extends/sustains the memory activation and thus helps to stabilize item-specific STM cell assemblies and their specificity. This effect may be called auto-consolidation, and it is an emergent feature of the plastic STM–LTM loop in our model. It occurs on a timescale governed by the unmodulated plasticity time constant (*κ* = *κ*_normal_, *τ_p_* = 5 s; Table 1). After a few seconds, the network has effectively stabilized and typically maintains a small set of three to four activated long-term memories. The closed STM–LTM loop thus constitutes a functional multi-item WM.

A crucial feature of any WM system is its flexibility, and Figure 4*B* highlights an example of rapid updating. The maintained set of activated memories can be weakened by stimulating yet another set of input memories. Generally speaking, earlier items are reliably displaced from active maintenance in our model if activation of the new items is accompanied by the same transient elevation of plasticity (*k_p_*/*k*_normal_; Table 1) used during the original encoding of the first five memories.

In line with the earlier results by Fiebig and Lansner (2017), cued activation can usually still retrieve previously maintained items. The rate of decay for memories outside the maintained set depends critically on the amount of noise in the system, which erodes the learned associations between STM and LTM neurons as well as STM cell assemblies. We note that such activity-dependent memory decay is substantially different from time-dependent decay, as shown by Mi et al. (2017).

### Multimodal, multi-item working memory

Next, we explore the ability of the closed STM–LTM loop system to flexibly bind coactive pairs of long-term memories from different modalities (LTMa and LTMb, respectively). As both LTM activations trigger cells in STM via FF projections, a unique joint STM cell assembly with shared pattern selectivity is created. Forward activations include excitation and inhibition, and combine nonlinearly with each other (Materials and Methods) and with prior STM content.

**Figure 5.**
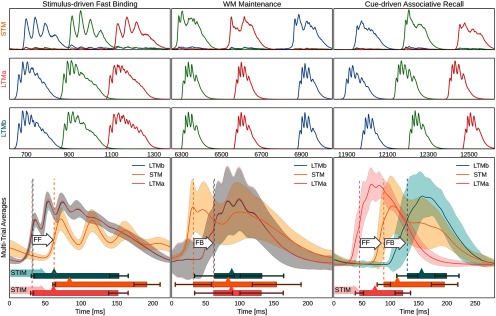
Population firing rates of networks and memory-specific subpopulations during three different modes of network activity. Top half, Exemplary activation of three memories (blue, green, and red, respectively) in STM (first row), LTMa (second row), and LTMb (third row) during the following three different modes of network activity: the initial association of pairs of LTM memory activations in STM (left column), WM maintenance through spontaneous STM-paced activations of bound LTM memory pairs (middle column), and cue-driven associative recall of previously paired stimuli (right column). Bottom half, Multitrial peristimulus activity traces from the three cortical patches across 100 trials (495 traces, as each trial features five activated and maintained LTM memory pairs and very few failures of paired activation). Shaded areas indicate an SD from the underlying traces. Vertical dashed lines denote mean onset of activity for each network, as determined by activation half-width (Materials and Methods), also denoted by a box underneath the traces. Error bars indicate an SD from activation onset and offset. Mean peak activation is denoted by a triangle on the box, and shaded arrows to the left of the box denote targeted pattern stimulation of a network at time 0. As there are no external cues during WM maintenance (i.e., the delay period), we use detected STM activation onset to align firing rate traces of 5168 STM-paced LTM reactivations across trials and reactivation events for averaging. White arrows annotate FF and FB delay, as defined by respective network onsets. Extended Data Figure 5-1 further illustrates the subsampled spiking activity in the three networks, during the multimodal LTM binding task.

Figure 5 illustrates how this new index then supports WM operations, including delay maintenance through STM-paced coactivation events and stimulus-driven associative memory pair completion. The three columns of Figure 5 illustrate the following three fundamental modes of the closed STM–LTM loop: stimulus-driven encoding, WM maintenance, and associative recall. The top three rows show sampled activity of a single trial, whereas the bottom row shows multitrial averages.

10.1523/ENEURO.0374-19.2020.f5-1 Figure 5-1 Spiking activity in the three networks, during the multimodal LTM binding task. Subsampled spike raster of the layer 2/3 population in a hypercolumn of STM (top), and five coding minicolumns in LTMa (second row) and LTMb (third row) respectively during plasticity-modulated stimulation (i.e., encoding), subsequent maintenance, and associative cued recall of five paired LTM patterns (orange, purple, blue, green, red). Minicolumns are separated by gray horizontal lines. STM spikes are colored according to the dominant memory pair selectivity in each cell. LTM Spikes are colored according to the memory pair specificity of each cell in slightly shifted hues to illustrate that LTMa and LTMb code for different, but associated memories. Bottom, Stimuli to LTM and modulation of plasticity. Note the cued recall of all five memories at the end. Download Figure 5-1, TIF file.

During stimulus-driven fast binding, we coactivate memories from both LTMs by brief 50 ms cues that trigger activation of the corresponding memory patterns. The average of peristimulus activations reveals 45 ± 7.3 ms LTM attractor activation delay, followed by 43 ± 7.8 ms FF delay (about half of which is explained by axonal conduction delays due to the spatial distance between LTM and STM) from the onset of the LTM activations to the onset of the input-specific STM response (Fig. 5, top left, bottom left).

**Figure 6.**
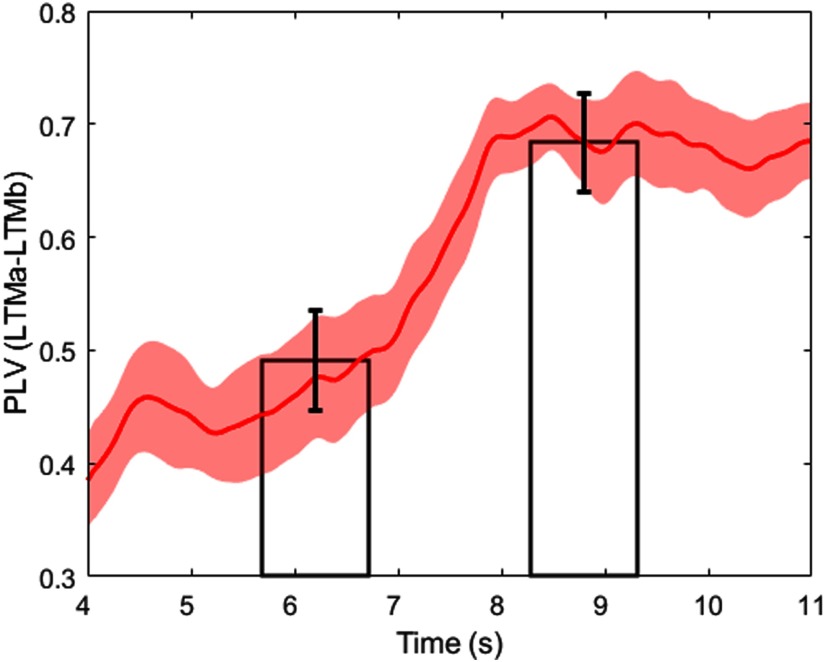
Gamma band PLV between LTMa and LTMb during WM maintenance. PLV is estimated using sliding window of size 0.5 s (the period between 4 and 12 s is shown). Two bars demonstrate the average gamma-band PLV over the first (4–8 s) and the second part (8–12 s) of the WM maintenance period. Shaded area and error bars correspond to the SEM calculated over *n* = 25 trials.

During WM maintenance, a 10 s delay period, paired LTM memories reactivate together. The onset of these paired activations is a lot more variable than during cued activation with an FB delay mean of 41.5 ± 15.3 ms, mostly because the driving STM activations are of variable size and strength. Over the course of the maintenance period, the oscillatory dynamics of the LTMs changes. In particular, LFP spectral power as well as coherence between LTMs in the broad gamma band (30–80 Hz) increases (*p* < 0.001 for each of two permutation tests comparing average spectral power/coherence in the gamma band between two intervals during the delay period: 4–8 s and 8–12 s; *n* = 25 trials). To study the fast oscillatory dynamics of the LFP interactions between LTMs during the WM maintenance, mediated by STM, we follow up the coherence analysis and examine the gamma phase synchronization effect using PLV with 0.5 s sliding window (see Materials and Methods). It appears that the gamma phase coupling also increases during the second part of the WM maintenance period (*p* < 0.001 in the analogous permutation test, as described above; Fig. 6).

Following the maintenance period, we test the ability of the memory system for bimodal associative recall. To this end, we cue LTMa, again using a targeted 50 ms cue for each memory, and track the systems response across the STM–LTM loop. We compute multitrial averages of peristimulus activations during recall testing (Fig. 5, bottom right). Following cued activation of LTMa, STM responds with the related joint cell assembly activation as the input is strongly correlated to the learned inputs, as a result of the simultaneous activation with LTMb earlier on. Similar to the mnemonic function of an index, the completed STM pattern then triggers the associated memory in LTMb through backprojections. STM activation now extends far beyond the transient activity of LTMa because STM recurrent connectivity and the STM–LTMb recurrence re-excites it. The temporal overlap between associated LTMa and LTMb memory activations peaks at ∼125 ms after the initial stimulus to LTMa.

### Network power spectra and the nonassociative control case

**Figure 7.**
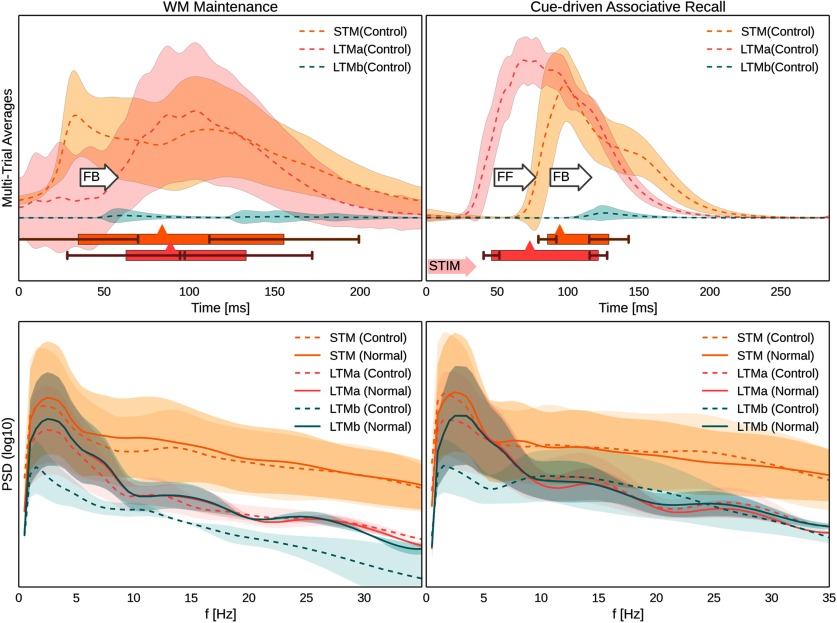
Nonassociative control case and power spectral analysis. Top half, Multitrial peristimulus activity traces from the three cortical patches across 25 trials following WM-encoded LTMa activations as before, but without associated LTMb memory activations. Shaded areas indicate a SD from the underlying traces. Activation half-widths (Materials and Methods) denoted by a box underneath the traces. Error bars indicate an SD from activation onset and offset. Mean peak activation is denoted by a triangle on the box, and shaded arrows to the left of the box denote targeted pattern stimulation of LTMa at time 0. As there are no external cues during WM maintenance (called the delay period), we use detected STM activation onset to align firing rate traces of 406 STM-paced LTMa reactivations across trials and reactivation events for averaging. There is no evidence of associated LTMb activations in the control case (only small increases in spike rate variability). White arrows annotate FF and FB delay, as defined by respective network onsets. Bottom half, Power spectral density of synthesized LFPs estimated over the maintenance (left) and recall (right) periods for STM and both LTMs in two cases: with (solid lines) and without (dashed line; control case) associated LTMb memory activations. Please note the log scale. Shaded areas correspond to the SD of the mean PSD over 25 trials. The decrease in theta- and gamma-band power observed during the maintenance (left) and recall (right) periods in the LTMb in the control case is due to lack of memory pattern reactivations in LTMb as they are not associated with LTMa via STM.

**Figure 8.**
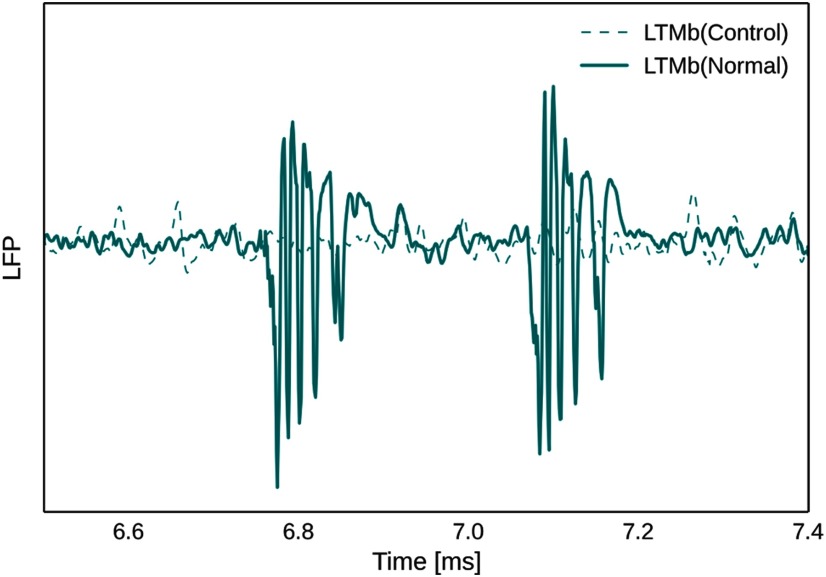
Exemplary recording of the LFP signal in LTMb following two cued activations of LTMa after learning and maintenance of associative LTMa–LTMb memory pairs (normal) or nonassociative LTMa memories without concurrent LTMb activation (control). While the LFP signal shows clear activation of associated LTMb items, LTMa-specific cues do not elicit memory activations in LTMb in the control case.

Figure 7 (top) shows multitrial peristimulus/periactivation activity traces for a control task, where learned and maintained LTMa items are not associated with concurrent LTMb activations. LTMa items are still encoded in STM, maintained over the delay, and recalled by specific cues, but LTMb now remains silent throughout the maintenance period (Fig. 7, top left) and, as expected, does not show any evidence of memory activation following LTMa-specific cues during recall testing (Fig. 7, top right, Fig. 8, LFP signal). The logarithmic power spectra (Fig. 7, bottom) show a noticeable difference between the normal associative and the nonassociative control trials. The latter displays a significant drop in LTMb power across the board, particularly during the maintenance period. This can be explained by the overall lower number of memory reactivations in STM during the nonassociative control task (2.58 ± 0.28 vs 1.62 ± 0.47 reactivations/s).

### Top-down and bottom-up delays

**Figure 9.**
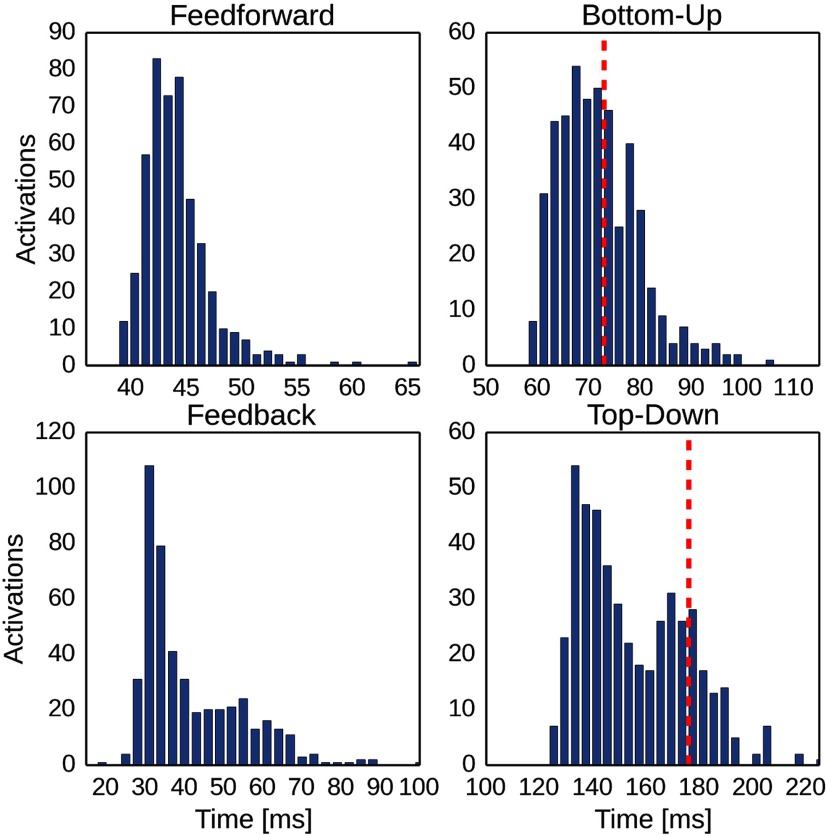
Comparison of key activation delays during associative recall in model and experiment following a cue to LTMa. Top left, Feedforward delay distribution in the model, as defined by the temporal delay between LTMa onset and STM onset (Fig. 4, bottom right). Top right, Bottom-up delay distribution in the model, as defined by the temporal delay between stimulation onset and LTMa peak activation. The red line denotes the mean bottom-up delay, as measured by Tomita et al. (1999). Bottom left, Feedback delay distribution in the model, as defined by the temporal delay between STM onset and LTMb onset (Fig. 4, bottom-right, measured by half-width). Bottom right, Top-down delay distribution in the model, as defined by the temporal delay between stimulation onset and LTMb peak activation. The red line denotes the mean bottom-up delay, as measured by Tomita et al. (1999). Model delays were averaged over 100 trials with five paired stimuli each.

We collected distributions of FF and FB delays during associative recall (Fig. 9). To facilitate a more immediate comparison with biological timing data, we also computed the bottom-up and top-down response latencies of the model in analogy to Tomita et al. (1999). Their study explicitly tested widely held beliefs about the executive control of PFC over ITC in memory retrieval. To this end, they identified and recorded neurons in ITC of monkeys trained to memorize several visual stimulus–stimulus associations. They used a posterior-split brain paradigm to cleanly disassociate the timing of the bottom-up (contralateral stimuli) and top-down (ipsilateral stimuli) responses in 43 neurons that were significantly stimulus selective in both conditions. They observed that the latency of the top-down response (178 ms) was longer than that of the bottom-up response (73 ms).

Our simulation is analogous to this experimental setup with respect to some key features, such as the spatial extent of memory areas (STM/dlPFC, ∼289 mm^2^) and interarea distances (40 mm cortical distance between PFC and ITC). These measures heavily influence the resulting connection delays and the time needed for information integration. In analogy to the posterior-split brain experiment, the LTMa and LTMb in our model are unconnected. However, we now have to consider them as ipsilateral and contralateral visual areas in ITC. The display of a cue in one hemifield in the experiment then corresponds to the LTMa-sided stimulation of an associated memory pair in the model. This arrangement forces any LTM interaction through STM (representing PFC) and allows us to treat the cued LTMa memory activation as a bottom-up response, whereas the much later activation of the associated LTMb representation is related to the top-down response in the experimental study. Figure 9 shows the distribution of these latencies in our simulations, where we also marked the mean latencies measured by Tomita et al. (1999). The mean of our bottom-up delay (72.9 ms) matches the experimental data (73 ms), whereas the mean of the broader top-down latency distribution (155.2 ms) is a bit lower than that in the monkey study (178 ms). Only 31 % (48 ms) of the top down delay (155.2 ms) was explained by the spatial distance between networks, as verified by a simulated model with 0 mm distance between networks.

## Discussion

In this work, we have proposed and studied a novel theory for WM that rests on the dynamic interactions between STM and LTM stores enabled by fast synaptic plasticity. In particular, it hypothesizes that activity in parietotemporal LTM stores targeting PFC via fixed or slowly plastic and patchy synaptic connections triggers an activity pattern in PFC, which then gets rapidly encoded by means of fast Hebbian plasticity to form a cell assembly. Equally plastic backprojections from PFC to the LTM stores are enhanced as well, thereby associating the formed PFC “index” specifically with the active LTM cell assemblies. This rapidly but temporarily enhanced connectivity produces a functional WM superassembly (a distributed constellation of cell assemblies) capable of encoding and maintaining multiple individual LTM items (i.e., bringing these LTM representations “online”) and forming novel associations within and between several connected LTM areas and modalities. The PFC cell assemblies themselves do not encode much information but act as indices of LTM stores, which contain additional information that is also more permanent. The underlying highly plastic connectivity and thereby the WM itself is flexibly remodeled and updated as new incoming activity gradually overwrites previous WM content. How quickly working memory is established after the initial encoding period, critically depends on plasticity modulation, network size, and overall activity. Our model does not address other important aspect of WM (e.g., the task relevance filtering and attentional gating involving upstream subcortical structures like basal ganglia and amygdala; O’Reilly and Frank, 2006; McNab and Klingberg, 2008).

We have studied the functional and dynamical implications of this theory by implementing and evaluating a special case of a biologically plausible large-scale spiking neural network model representing PFC reciprocally connected with two LTM areas (e.g., visual and auditory) in temporal cortex. We have shown how a number of single LTM items can be encoded and maintained online, and how pairs of simultaneously activated items can become jointly indexed and associated. Activating one pair member now also activates the other one indirectly via PFC with a short latency. We have further demonstrated that this kind of WM can readily be updated, such that as new items are encoded, old items are fading away, whereby the active WM content is replaced. Notably, unlike in our model, in a biological brain many long-range connections exist between LTM areas, and they will significantly influence the sequence of recalled items.

Recall dynamics in the presented model are in most respects identical to a previous cortical associative memory model (Lansner, 2009) and also to that of single-item persistent activity WM models (Camperi and Wang, 1998). Any activated memory item, whether randomly or specifically triggered, is subject to known and previously well characterized associative memory dynamics, such as pattern completion, rivalry, bursty reactivation dynamics, and oscillations in different frequency bands (Lundqvist et al., 2010, 2013; Silverstein and Lansner, 2011; Herman et al., 2013). Moreover, sequential learning and recall could readily be incorporated (Tully et al., 2016). This could, for example, support encoding of sequences of items in WM rather than a set of unrelated items, resulting in reactivation dynamics reminiscent of, for instance, the phonological loop (Baddeley et al., 1998; Burgess and Hitch, 2006).

### Cortical indexing theory for WM

Our model draws inspiration from the hippocampal indexing theory (Teyler and DiScenna, 1986), originally proposed to account for the role of hippocampus in storing episodic memories (Teyler and Rudy, 2007). While there are key similarities, there are also a number of important differences. Similar to the hippocampus, PFC is well connected with association cortices to directly or indirectly influence activity across most of cortex (Pandya and Yeterian, 1991; Pandya and Barnes, 2019). Unlike the hippocampal indexing theory, which posits that such influence is not seen until the eventual recall, we propose and demonstrate in simulation that the creation of the PFC index has immediate effects on neocortical activity patterns, manifested as WM delay activity across widely distributed cortical areas (Tomita et al., 1999; Sreenivasan and D’Esposito, 2019).

In line with Teyler and Discenna (1986), we propose that the rapid encoding necessitated by indexing is enabled by transient dopaminergic modulation of plasticity among recurrently connected neurons and their FB projections onto cortex. As suggested for hippocampus, PFC does not store the sensory content of WM itself, but rather an index to task-relevant information carried by areas lower in the cortical hierarchy. As PFC integrates information across cortex (Miller and Cohen, 2001), the index becomes part of a temporary WM superassembly. Hippocampal indexing is largely seen as a process preceding cortical long-term consolidation, whereby associations between indexed areas eventually become independent of hippocampus. Our model makes no such claim for the role of PFC. On the contrary, WM function rests on the fluidly changing selectivity of PFC neurons, which would be hampered by strong LTP and slow processes of consolidation. Yet an intriguing possibility suggested by our quantitative model is that a hippocampal indexing network with a longer plasticity time constant operating analogously to our PFC model could support such a consolidation process by reinstating activity in cortical LTM areas.

### The case for Hebbian plasticity

The underlying mechanism of our model is fast Hebbian plasticity, not only in the intrinsic PFC connectivity, but also in the projections from PFC to LTM stores. The former has some experimental support (Volianskis and Jensen, 2003; Erickson et al., 2010; Park et al., 2014; Volianskis et al., 2015; Pradier et al., 2018), whereas the latter remains a prediction of the model. D1R activation by DA is strongly implicated in reward learning and synaptic plasticity regulation in the basal ganglia (Wickens, 2009). In analogy, we propose that D1R activation is critically involved in the synaptic plasticity intrinsic to PFC and its projections to LTM stores, which would also explain the comparatively dense DA innervation of PFC and the prominent WM effects of PFC DA level manipulation (Goto et al., 2010; Arnsten and Jin, 2014). In the model presented here, the parameter *κ* represents the level of DA–D1R activation, which in turn regulates synaptic plasticity. We typically increase κ temporarily (Table 1) in conjunction with stimulation of LTM and WM encoding, in a form of attentional gating. Excessive modulation limits WM capacity to one to two items, while less modulation diminishes the strength of cell assemblies beyond what is necessary for reactivation and LTM maintenance.

When the synaptic plasticity WM hypothesis was first presented and evaluated, it was based on synaptic facilitation (Mongillo et al., 2008; Lundqvist et al., 2011). However, such non-Hebbian plasticity is only capable of less specific forms of memory. Activating a cell assembly comprising a subset of neurons in an untrained STM network featuring such plasticity would merely facilitate all outgoing synapses from active neurons. Likewise, an enhanced elevated resting potential resulting from intrinsic plasticity would make the targeted neurons more excitable. In neither case would there be any coordination of activity specifically within the stimulated cell assembly. Thus, if superimposed on an existing LTM, such forms of plasticity may well contribute to WM, but they are by themselves not capable of supporting encoding of novel memory items or the multimodal association of already existing ones. In contrast, previous work by Fiebig and Lansner (2017) showed that fast Hebbian plasticity similar to short-term potentiation (Erickson et al., 2010) allows effective one-shot encoding of novel STM items. In the extended model proposed here, PFC can additionally bind and bring online existing but previously unassociated LTM items across multiple modalities by means of the same kind of plasticity in backprojections from PFC to parietotemporal LTM stores.

On a side note, this implementation of fast Hebbian plasticity reproduces a remarkable aspect of short-term potentiation or labile LTP: it decays in an activity-dependent manner rather than with time (Volianskis and Jensen, 2003; Volianskis et al., 2015; Pradier et al., 2018). Although we used the BCPNN learning rule to reproduce these effects, we expect that other Hebbian learning rules allowing for neuromodulated fast synaptic plasticity could give comparable results.

### Experimental support and testable predictions

Our model has been built from available relevant microscopic data on neural and synaptic components as well as the modular structure and connectivity of selected cortical areas in macaque monkey. Its sparse corticocortical long-range connectivity is compatible with neuroanatomical data and can add specific predictions of the nature of this connectivity. The network so designed generates a well organized macroscopic dynamic working memory function, which can be interpreted in terms of manifest behavior and validated against cognitive experiments and data. Our model provides a powerful tool to investigate and examine the link between microscopic and macroscopic level processes and data. It suggests novel mechanistic hypotheses and inspiration for planning and performing experiments that can develop further the model, or potentially falsify it.

Unfortunately, the detailed neural processes and dynamics of our new model are not easily accessible experimentally as they are intrinsically expressed at multiple scales (e.g., mesoscopic field potentials and population spiking at macroscopic spatial scales). In consequence, it is difficult to find direct and quantitative results to validate the model. To our knowledge, no other WM model of comparable detail has been reported. On the one hand, some recent models that explain WM activity through the long-range interactions of STM and LTM systems (Bouchacourt and Buschman, 2019) lack defensible constraints on the density of the long-range projections involved. On the other hand, a more complete cortical model by Schmidt et al. (2018), accounting for available corticocortical connectivity data from layer-specific retrograde tracing experiments (Markov et al., 2014), was not concerned with any concrete aspects of cognitive function, such as working memory. We specifically tested extremely sparse and plastic corticocortical connectivity and demonstrated its effectiveness in indexing working memory.

In analyzing our resulting bottom-up and top-down delays, we drew an analogy to a split-brain experiment (Tomita et al., 1999) because of its clean experimental design (even controlling for subcortical pathways) and found similar temporal dynamics in our highly subsampled cortical model. The timing of interarea signals also constitutes a testable prediction for multimodal memory experiments. Furthermore, reviews of intracranial as well as electroencephalography (EEG) recordings conclude that theta band oscillations play an important role in long-range communication during successful memory retrieval (Sauseng et al., 2004; Johnson and Knight, 2015). With respect to theta band oscillations in our model, we have shown that STM leads the LTM networks during maintenance, engages bidirectionally during recall (due to the STM–LTM loop), and lags during stimulus-driven encoding and LTM activation, reflecting experimental observations (Anderson et al., 2010). These effects are explained by our model architecture, which imposes delays due to the spatial extent of networks and their distances from each other. Fast oscillations in the broad gamma band, often nested in the theta cycle, are strongly linked to local processing and activated memory items in our model, also matching experimental findings (Canolty and Knight, 2010; Johnson and Knight, 2015). Local frequency coupling is abundant with significant phase–amplitude coupling (Fig. 3*B*), and was well characterized in related models (Herman et al., 2013).

The most critical requirement and thus prediction of our theory and model is the presence of fast Hebbian plasticity in the PFC backprojections to parietotemporal memory areas. Without such plasticity, our model cannot explain the necessary STM–LTM binding. This plasticity is likely to be subject to neuromodulatory control, presumably with DA and D1R activation involvement. Since short-term potentiation decays with activity, a high noise level could be an issue since it could shorten WM duration (see The case for Hebbian plasticity). The evaluation of this requirement is hampered by lack of experimental evidence and characterization of the synaptic plasticity in corticocortical projections.

One of the neurodynamical manifestations of the fast associative plasticity in the PFC backprojections is a functional coupling between LTM stores. Importantly, this long-range coupling in our model is mediated by the PFC network alone, as manifested during the delay period free of any external cues, and is reflected in the synchronization of fast gamma oscillations. Although the predominant view has been that gamma is restricted to short distances, there is growing evidence for cortical long-distance gamma phase synchrony between task-relevant areas as a correlate of cognitive processes (Tallon-Baudry et al., 1998; Doesburg et al., 2008) including WM (Palva et al., 2010). In this regard, our model generates even a more specific prediction about the notable temporal enhancement of gamma phase coupling over the delay period, which could be tested with macroscopic human brain recordings (e.g., EEG or MEG), provided that a WM task involves a sufficiently long delay period.

Finally, our model suggests the occurrence of a double peak of frontal network activation in executive control of multimodal LTM association (Figs. 4*C*, 5, STM population activity during WM maintenance). The first one originates from the top-down control signal itself, and the second one is a result of corticocortical reentry and a successful activation of one or more associated items in LTM. As such, the second peak should also be correlated with successful memory maintenance or associative recall.

### Possible role for fast Hebbian plasticity in variable binding

The “binding problem” is a classical and extensively studied problem in perceptual and cognitive neuroscience (Zimmer et al., 2012). Binding occurs in different forms and at different levels, from lower perceptual to higher cognitive processes (Reynolds and Desimone, 1999; Zimmer et al., 2006; Zmigrod et al., 2014).

**Figure 10.**
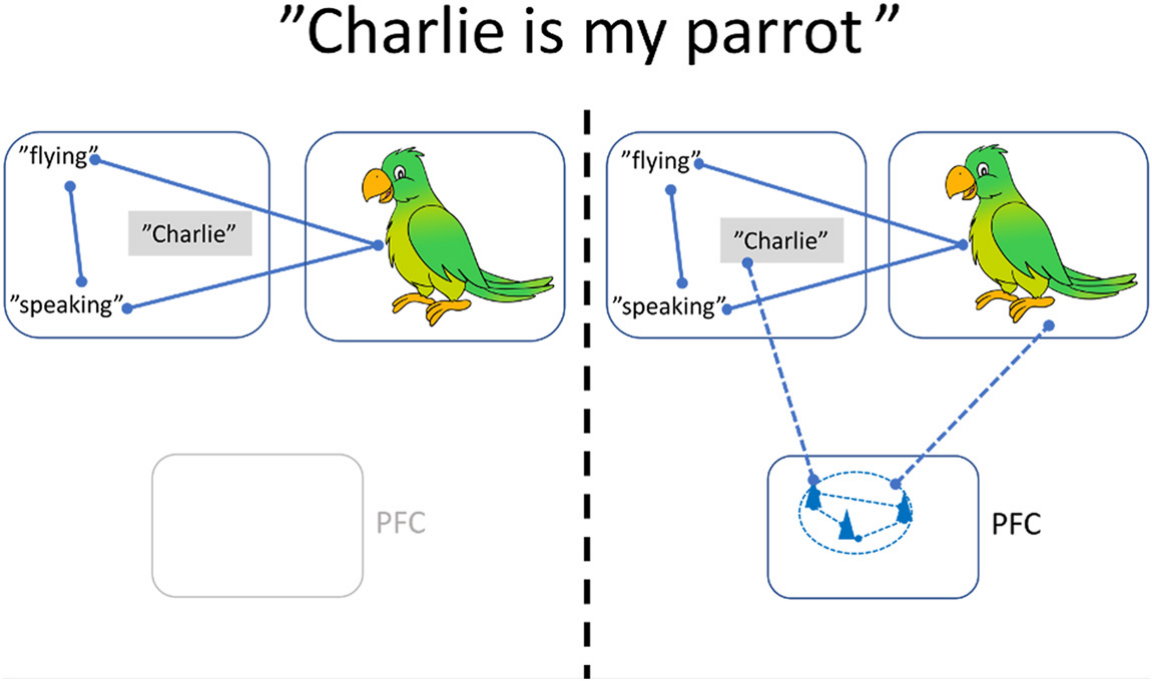
Variable value binding via index in PFC. Initially, the multimodal representation of “parrot” exists in LTM comprising symbolic and subsymbolic components side by side with “Charlie” as a representation of a proper name. It is hypothesized here that when someone states that “Charlie is my parrot,” the name “Charlie” is temporarily and reciprocally bound to the parrot representation via PFC, mediated by fast Hebbian plasticity. Pattern completion effect now allows “Charlie” to trigger the entire assembly and, analogously, makes “flying” or the sight of a given parrot trigger “Charlie.” If important enough or repeated a couple of times this association could consolidate in LTM.

Variable binding is a cognitive kind of neural binding in the form of a temporary variable–value association of items previously not connected by earlier experience and learning (Cer and O’Reily, 2012; Garnelo and Shanahan, 2019). A simple special case is the association of a mathematical variable and its value “The value of *x* is 2” (i.e., *x* = 2) or of an object and its proper name as in “Charlie is my parrot” (Fig. 10). This and other more advanced forms of neural binding are assumed to underlie complex functions in human cognition including logical reasoning and planning (Pinkas et al., 2012), but have been a challenge to explain by neural network models of the brain (van der Velde and de Kamps, 2015; Legenstein et al., 2016). Work is in progress to uncover how such variable binding mechanisms can be used in neuro-inspired models of more advanced human logical reasoning (Pinkas et al., 2013).

Based on our WM model, we propose that a PFC/STM index mediated by fast Hebbian plasticity provides a neural mechanism that could explain such variable binding. The joint index formed in PFC during presentation of a name–value pair serves to temporarily bind the corresponding representations. The value could be multimodal and include symbolic as well as subsymbolic components. Turning to Figure 5 above, imagine that one of the LTMa patterns represents the image of a parrot and one pattern in LTMb represents the proper name “Charlie.” If someone says “Charlie is my parrot,” these previously not associated items are rapidly bound via a joint PFC index. While this short-term connectivity remains, the name “Charlie” will trigger the internal object representation of a parrot, and seeing a parrot will trigger the name “Charlie” with the dynamics shown in the right-most panels of Figure 5 Flexible updating of the PFC index (Fig. 4) will avoid confusion even if in the next moment my neighbor shouts “Charlie” to call his dog, also named Charlie. If important enough or repeated a number of times, this association could further consolidate in LTM.

### Conclusions

We have formulated an indexing theory for cortical WM and tested it by means of computer simulations, which demonstrated the versatile WM properties of a large-scale spiking neural network model implementing key aspects of the theory. Our model provides a new mechanistic understanding of WM with distributed cortical correlates and variable binding phenomena, which connects microscopic neural processes with macroscopic observations and cognitive functions in a way that only computational models can do. While we designed and constrained this model based on macaque data, the theory itself is quite general, and we expect our findings to apply also to mammals, including humans, commensurate with changes in key model parameters (e.g., cortical distances, axonal conductance speeds). Many aspects of WM function remain to be tested and incorporated (e.g., its close interactions with basal ganglia; O’Reilly and Frank, 2006).

WM dysfunction has an outsized impact on mental health, intelligence, and quality of life. Progress in mechanistic understanding of its function and dysfunction is therefore very important for society. We hope that our theoretical and computational work provides inspiration for experimentalists to scrutinize the theory and model, especially with respect to neuromodulated fast Hebbian synaptic plasticity and large-scale network architecture and dynamics. Only in this way can we get closer to a more solid understanding and theory of WM, and position future computational research appropriately even in the clinical and pharmaceutical realm.

10.1523/ENEURO.0374-19.2020.ed1 Extended Data 1 We use the NEST simulator (Gewaltig and Diesmann, 2007) version 2.2 for our simulations (RRID: SCR_002963), running on a Cray XC-40 Supercomputer of the PDC Centre for High Performance Computing. The custom-build spiking neural network implementation of the BCPNN learning rule for MPI-parallelized NEST is freely available on github: https://github.com/Florian-Fiebig/BCPNN-for-NEST222-MPI and included in the extended data. Further, the model is also available on ModelDB (https://modeldb.yale.edu/257610). The depositories have README files, commented code, and we are happy to help individual researchers with using the BCPNN synapse model. We here briefly describe the installation procedure of the main BCPNN synapse model on the Git repository, or in the included zip file. After extracting the main folder BCPNN-for-NEST222-MPI-master in [PATH]. The compile script compile-module.sh switches the compiler to GNU, and adds Python2.7 and NEST 2.2.2 to the environment on a Cray XC-40 via the following: module swap PrgEnv-cray PrgEnv-gnu module add python/2.7.13 site-python/2.7 module add nest/2.2.2-py27. This obviously depends on the exact supercomputer environment and available modules. The compile script automatically creates necessary bootstrap and build directories, but some adjustments might be necessary, particularly with respect to the NEST Simulator install path, specified in line 54. Next, the compile script is run on the custom module like this: sudo ./compile-module.sh module-100725/. Then in python: import nest nest.sr(‘([PATH]/BCPNN-for-NEST222-MPI-master/share/nest/sli) addpath’) nest.Install(‘[PATH]/BCPNN-for-NEST222-MPI-master/lib/nest/pt_module’) The model should now be available as ‘bcpnn_synapse’ in the list of NEST synapses: print nest.Models(‘synapses’). Download Extended Data 1, ZIP file.

## Synthesis

Reviewing Editor: Gustavo Deco, Universitat Pompeu Fabra Decisions are customarily a result of the Reviewing Editor and the peer reviewers coming together and discussing their recommendations until a consensus is reached. When revisions are invited, a fact-based synthesis statement explaining their decision and outlining what is needed to prepare a revision will be listed below. The following reviewer(s) agreed to reveal their identity: Niraj Desai.

Reviewer 1:

The authors explore and develop an indexing theory of working memory - that is the manuscript’s main contribution. To do so, they build an elaborate (maybe too elaborate) model of the relevant brain areas. They include minicolumns and hypercolumns, short-term depression, adaptive-exponential model neurons, two classes of interneurons, various types of (biologically-motivated) connectivity, and short-term Hebbian plasticity.

That last thing gives me some pause - as I don’t think presynaptic, NMDA-independent, transient LTP is well-established in cortex - and the way it’s included (Bayesian Confidence Propagation Neural Network) is rather obscure and requires the reader to look through earlier papers to get what they’re doing.

I would recommend two general things:

1) A better justification of short-term Hebbian plasticity. Even if this paper has gone out a little over the skis as far as experimental justification goes, it would still be worth exploring how much the details of short-term plasticity matter by varying the parameters systematically. It may well be that labile or transient LTP is important in neocortex, but that its effects turn on the detail of the implementation. Varying parameters is a way of accounting for that possibility.

2) For similar reasons, justifying the complexity of the model would be useful. The authors write that a parameter sensitivity analysis is beyond the scope of their work. Fair enough, but it would be useful to demonstrate that some of the choices (adaptive-exponential IF neurons, Tsodyks-Markram synaptic depression) are necessary and useful. From these results, it’s not clear that they are.

... and one specific thing:

3) Check the references carefully. For example, Eriksson et al. (Neuron, 2015) is an important one that is cited but not included in the reference list.

----

Reviewer 2:

Work presents a neural network computational model for the study of the mechanism of STM-LTM interactions in WM. The proposed model seems to be replicating several observed experimental results like the oscillations, coherence, and latency within and between these areas. The authors also state that the model can be used to study the interneuron dynamics in WK.

Authors state that the goal is to show robustness and flexibility of their model, however they do not provide a detailed parameter sensitivity analysis to prove its robustness (stated in lines 366-367). Robustness is proven by simply the observation that this complex model is based on models that were proven robust by their authors (this is not a statement I personally agree with). Authors refer to “rigorous testing not shown here in the paper”. But why not?

Here are some corrections:

- lines 34 and 35 the ref to Eriksson paper - the correct year is 2010

- line 169 - why sd of 15%? Why this is a good choice. Please explain.

- section STM architecture describes the structure of the model organization. But the reader would have a better understanding if an image of the layered structure is shown.

I found this manuscript “An Indexing Theory for Working Memory based on Fast Hebbian Plasticity, by Florian Fiebig, Pawel Herman, Anders Lansner

bioRxiv 334821; doi: https://doi.org/10.1101/334821” in preprint. I believe it is the same article like the one submitted here for review.
