# Supplementary figures and images for: An Indexing Theory for Working Memory Based on Fast Hebbian Plasticity
Source: eNeuro. 2020 Mar 19;7(2):ENEURO.0374-19.2020. doi: 10.1523/ENEURO.0374-19.2020 (PMC7189483; doi:10.1523/ENEURO.0374-19.2020)

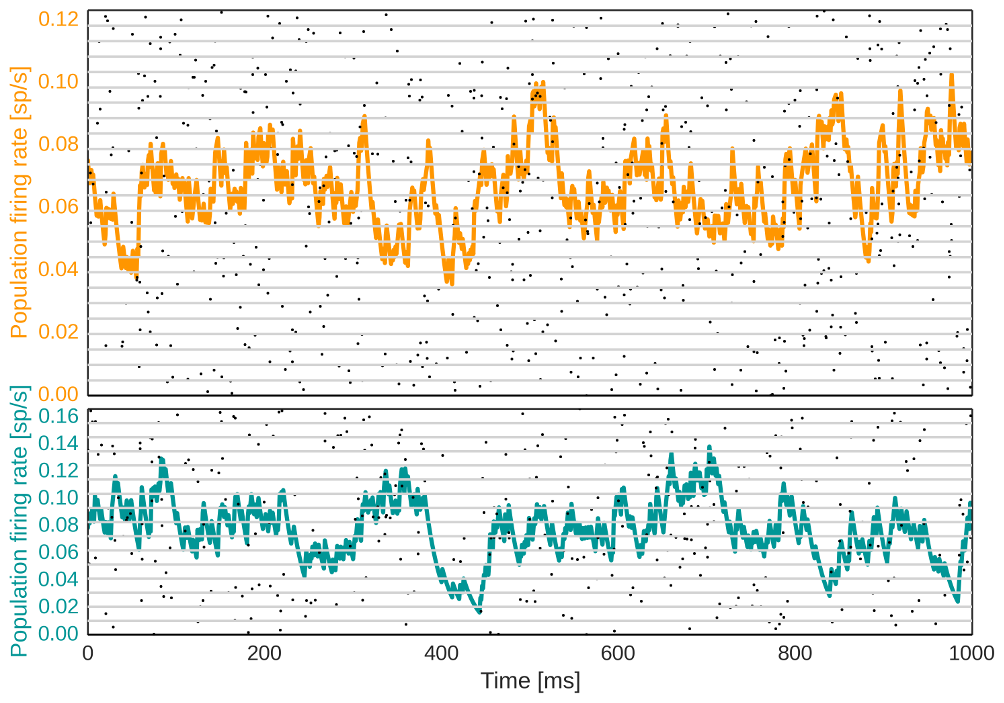

Supplement: Figure 3-1 — Basic network behavior in spike rasters and population firing rates under low input. The untrained networks STM (top) and LTM (bottom) feature low-rate, asynchronous activity (CV2 = 0.7 ± 0.2). The underlying spike raster shows layer 2/3 activity in each HC (separated by gray horizontal lines) in the simulated network. Download Figure 3-1, TIF file. [file enu-eN-NWR-0374-19-s01.tif]

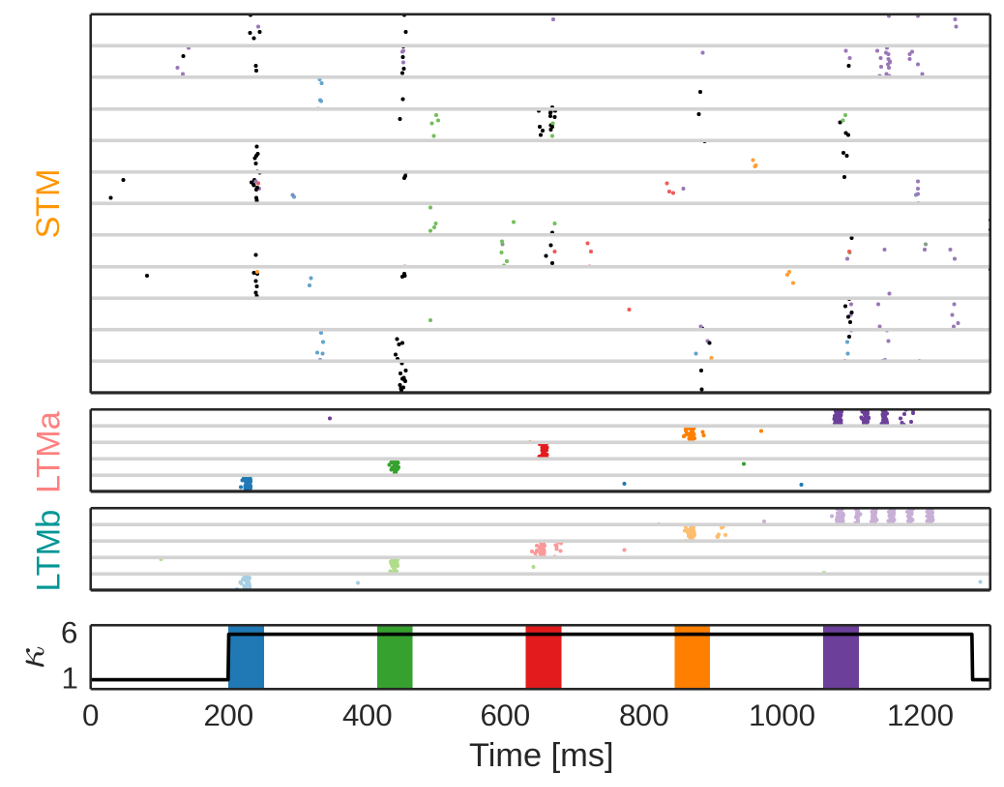

Supplement: Figure 3-2 — Network activity during plasticity-modulated stimulation with 20% spatial extent. Subsampled spike raster of the layer 2/3 population in a hypercolumn of STM (top), and five coding minicolumns in LTMa (second row) and LTMb (third row), respectively, during plasticity-modulated stimulation (i.e., encoding) of five paired LTM patterns. Without sufficient conduction delays, memory activations collapse into very brief bursts (with the exception of the last pattern here), and STM cannot effectively activate from, or subsequently encode, such brief activations (Fig. 3B,D). Download Figure 3-2, TIF file. [file enu-eN-NWR-0374-19-s02.tif]

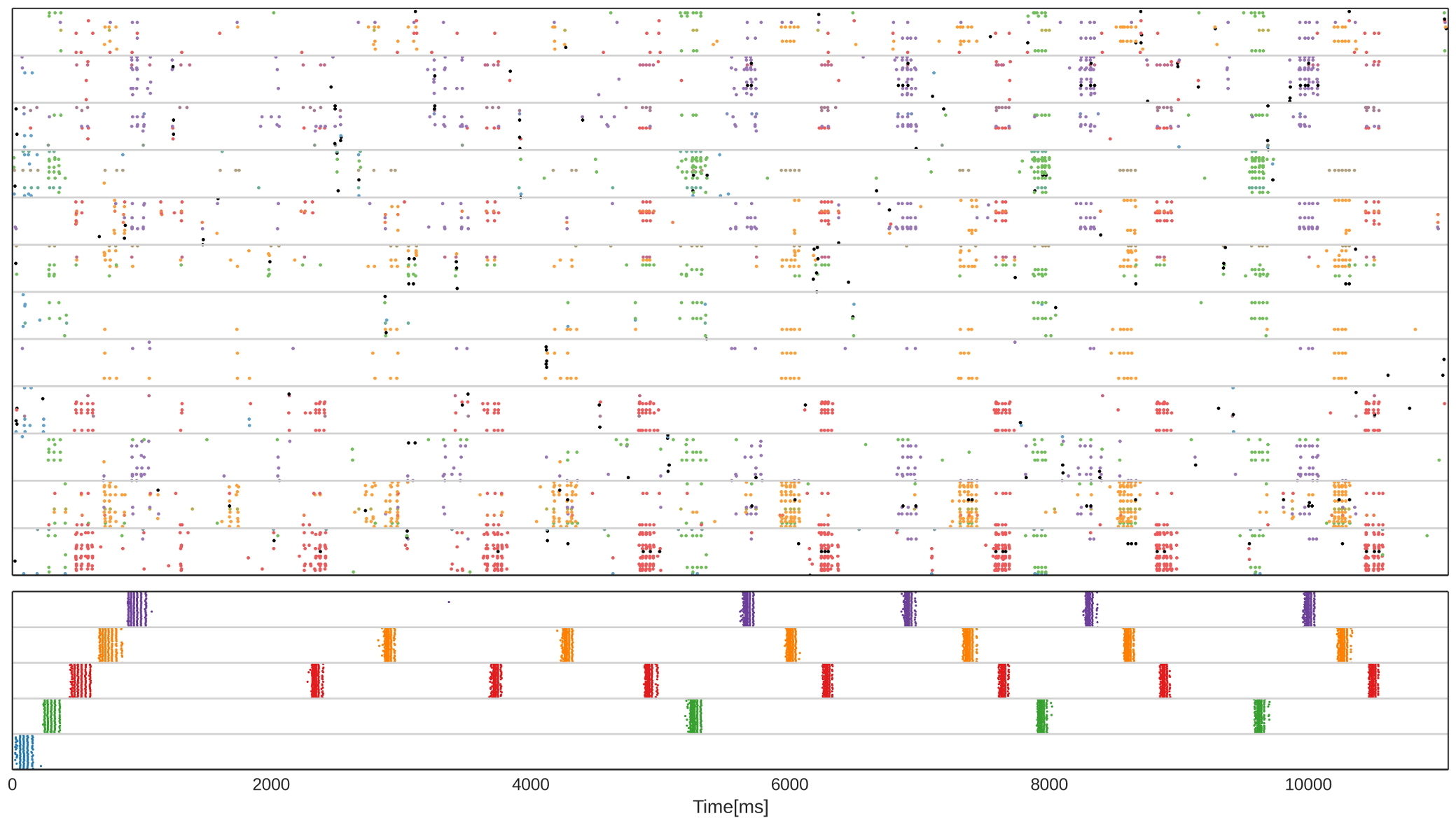

Supplement: Figure 4-1 — Encoding and feedback-driven reactivation of long-term memories. Subsampled spike raster of STM (top) and LTM (bottom) during encoding and subsequent maintenance of five memories (the first pattern is not maintained in this simulation). During the initial plasticity-modulated stimulation phase, five LTM memories are cued via targeted 50 ms stimuli (shown underneath). Plasticity of STM and its backprojections is modulated during this initial memory activation (Fig. 3D). Thereafter, a strong noise drive to STM causes spontaneous activations and plasticity-induced consolidation of pattern-specific subpopulations in STM. Backprojections reactivate associated LTM memories. Top, STM spike raster shows layer 2/3 activity in a single HC. MCs are separated by gray horizontal lines. STM spikes are colored according to each cell’s dominant LTM pattern-correlation, similar to Figure 2D. Bottom, LTM spike raster only shows the activity of five coding MC in a single LTM HC, but indicates the activation of distributed LTM memory patterns. LTM spikes are colored according to the pattern specificity of each cell. Download Figure 4-1, TIF file. [file enu-eN-NWR-0374-19-s03.tif]

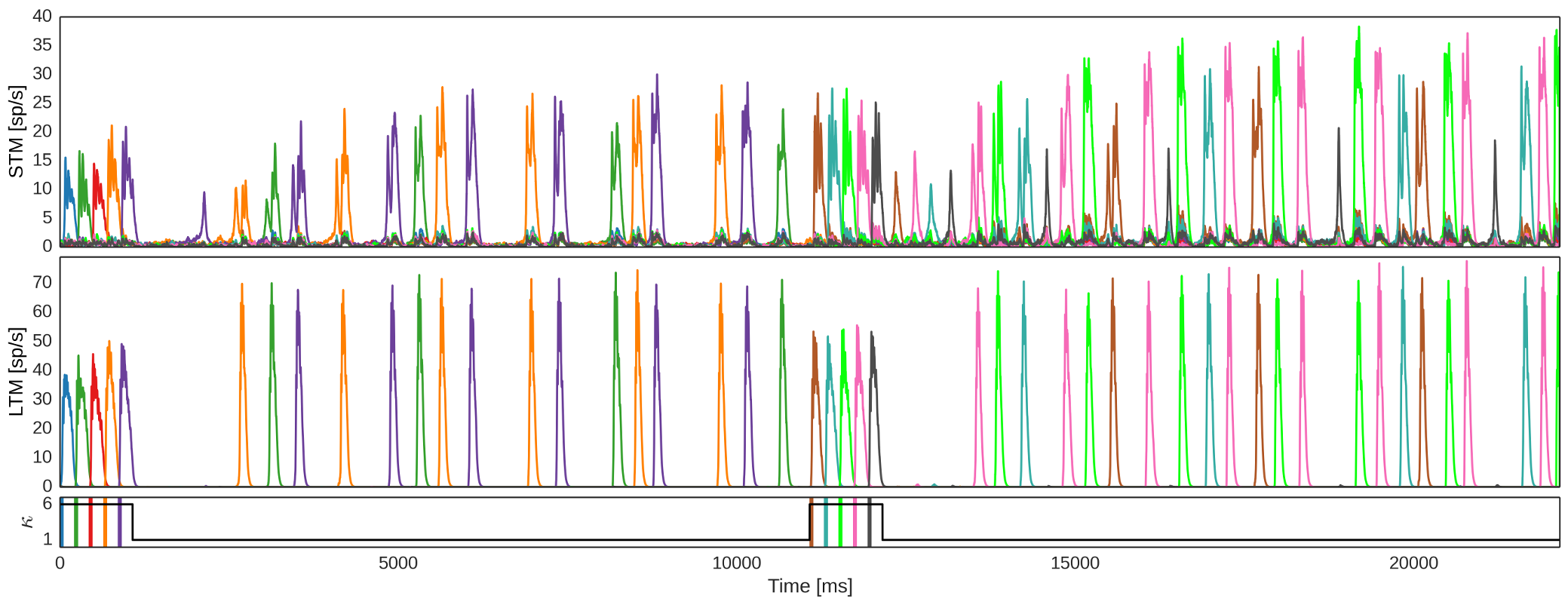

Supplement: Figure 4-2 — Spike rates during WM updating. Population firing rates of pattern-specific subpopulations in STM and LTM during encoding and subsequent maintenance of two sets of five LTM memories. After encoding and 10 s maintenance of the first set, WM contents are overwritten with the second set of memories, maintained thereafter in spontaneous reactivation events. Bottom: Stimuli to LTM and modulation of plasticity. Download Figure 4-2, TIF file. [file enu-eN-NWR-0374-19-s04.tif]

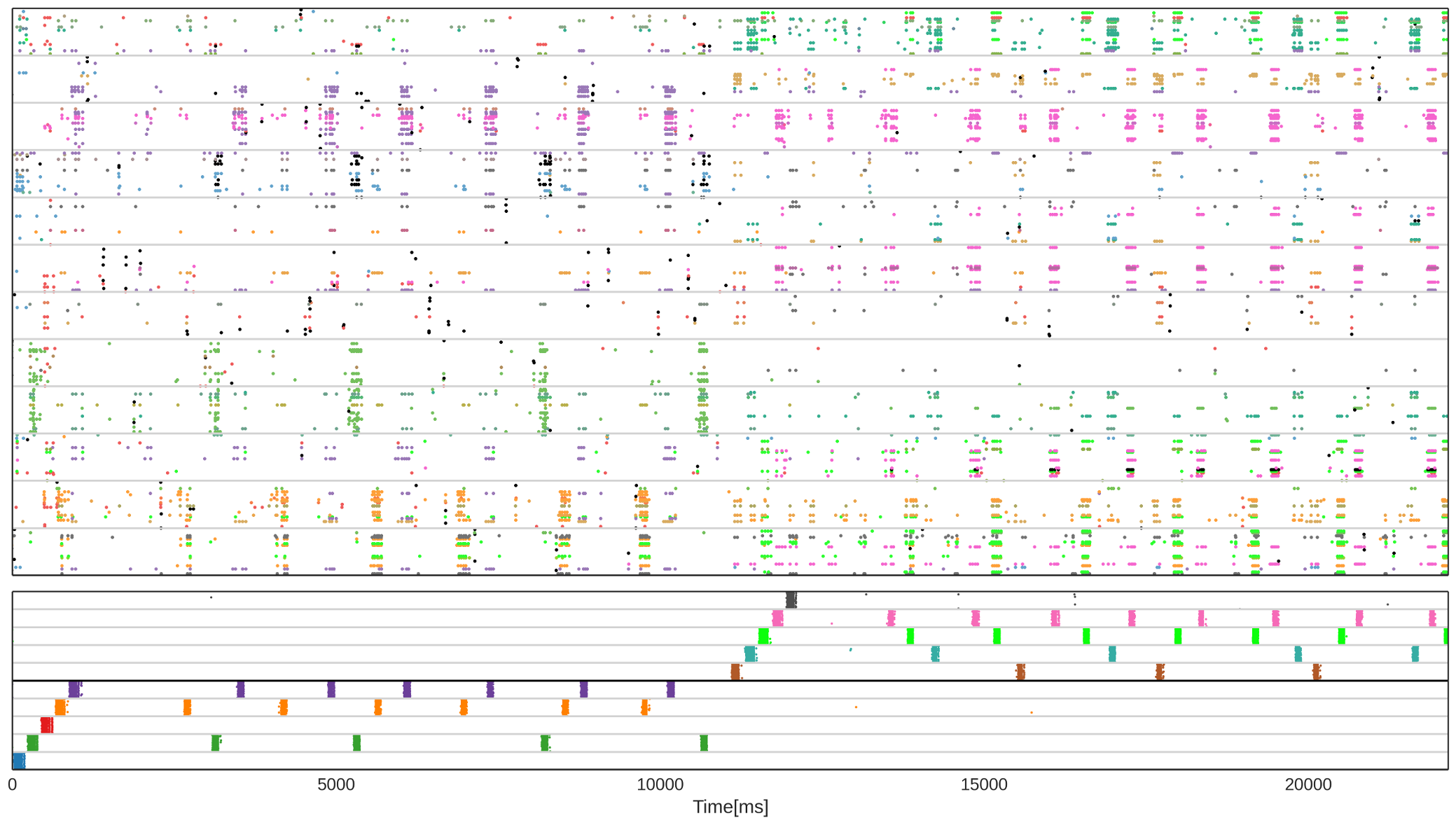

Supplement: Figure 4-3 — Spike raster during WM updating. Subsampled spike raster of the layer 2/3 population in a hypercolumn of STM (top) and LTM (bottom) respectively during encoding and subsequent maintenance of two sets of five LTM memories. STM spikes are colored according to each cells dominant pattern-selectivity. LTM spikes are colored according to the pattern-specificity of each cell. After encoding and 10 s maintenance of the first set, WM contents are overwritten with the second set of memories, maintained thereafter. Plasticity is temporarily boosted during the initial activation of LTM attractors (see preceding figure). Strong noise drive to STM causes spontaneous reactivations and consolidation of pattern-specific subpopulations in STM following each stimulation period. Download Figure 4-3, TIF file. [file enu-eN-NWR-0374-19-s05.tif]

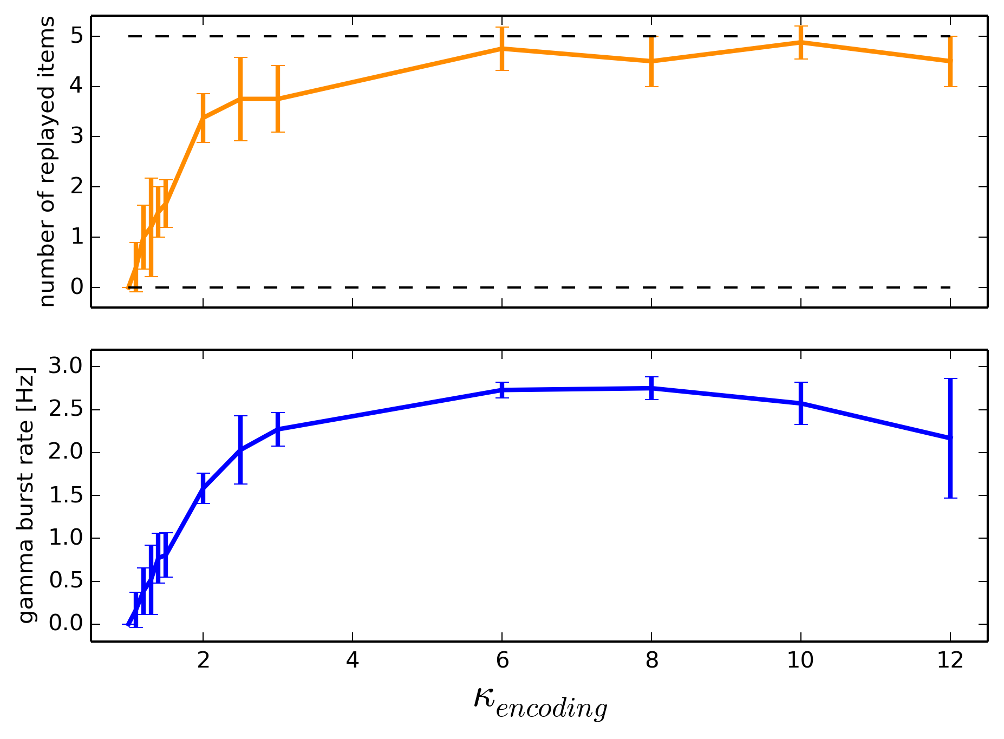

Supplement: Figure 4-4 — Sensitivity of WM delay activity to the plasticity modulation κencoding during encoding. The size of the set of actively maintained items (top) and the average rate of gamma burst events (bottom) over the 10 s delay period is reasonably stable around the operating point of κencoding = 6. Results are averaged from 84 simulation runs with varying amounts of plasticity modulation. Error bars denote the SE. Download Figure 4-4, TIF file. [file enu-eN-NWR-0374-19-s06.tif]

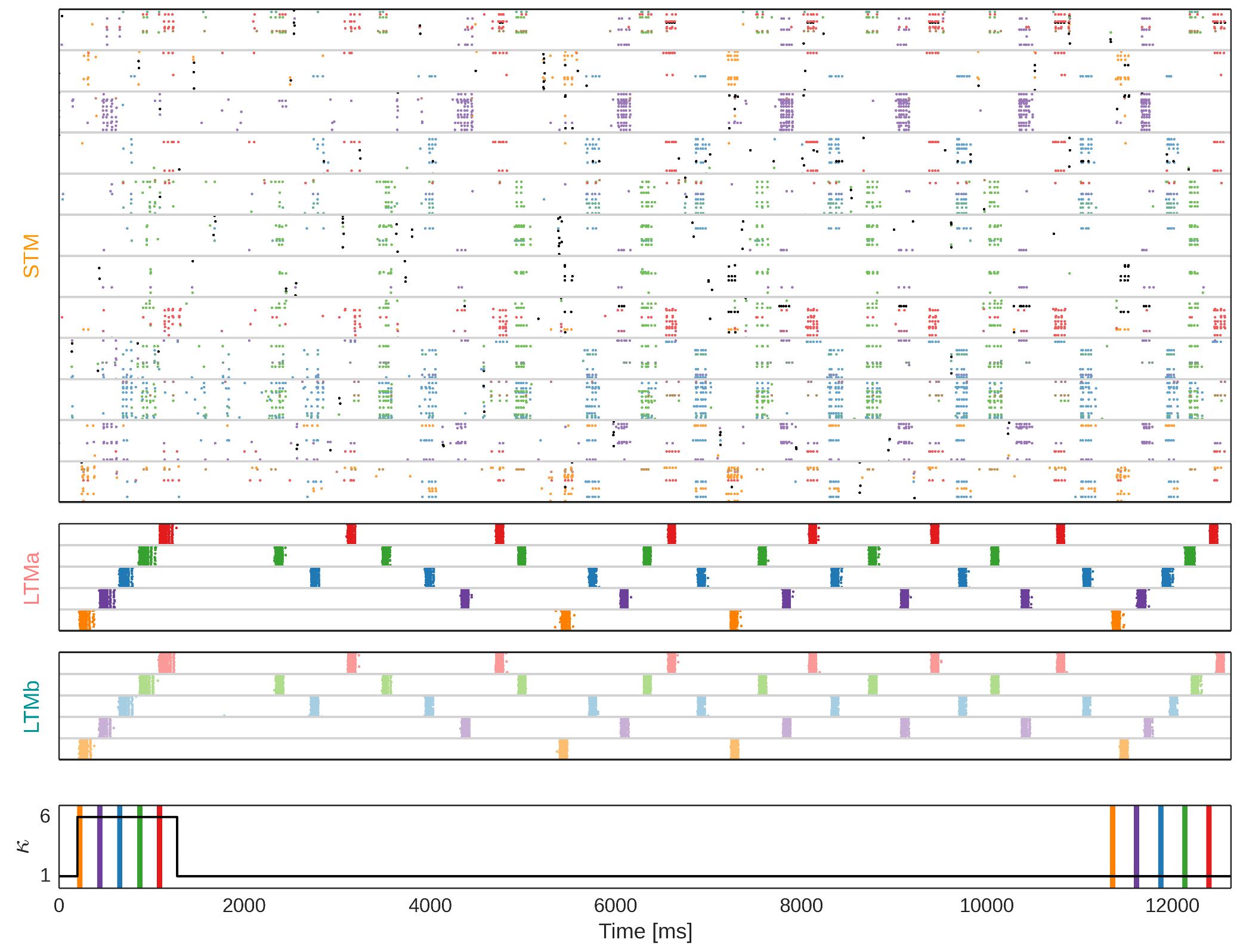

Supplement: Figure 5-1 — Spiking activity in the three networks, during the multimodal LTM binding task. Subsampled spike raster of the layer 2/3 population in a hypercolumn of STM (top), and five coding minicolumns in LTMa (second row) and LTMb (third row) respectively during plasticity-modulated stimulation (i.e., encoding), subsequent maintenance, and associative cued recall of five paired LTM patterns (orange, purple, blue, green, red). Minicolumns are separated by gray horizontal lines. STM spikes are colored according to the dominant memory pair selectivity in each cell. LTM Spikes are colored according to the memory pair specificity of each cell in slightly shifted hues to illustrate that LTMa and LTMb code for different, but associated memories. Bottom, Stimuli to LTM and modulation of plasticity. Note the cued recall of all five memories at the end. Download Figure 5-1, TIF file. [file enu-eN-NWR-0374-19-s07.tif]
